# Hormonal control of inflammatory responses

**DOI:** 10.1155/S0962935193000250

**Published:** 1993

**Authors:** J. Garcia-Leme, Sandra P. Farsky

**Affiliations:** Department of Pharmacology Institute of Biomedical Sciences University of São Paulo São Paulo SP 05508-900 Brazil

## Abstract

Almost any stage of inflammatory and immunological responses is affected by hormone actions. This provides the basis for the suggestion that hormones act as modulators of the host reaction against trauma and infection. Specific hormone receptors are detected in the reactive structures in inflamed areas and binding of hormone molecules to such receptors results in the generation of signals that influence cell functions relevant for the development of inflammatory responses. Diversity of hormonal functions accounts for recognized pro- and anti-inflammatory effects exerted by these substances. Most hormone systems are capable of influencing inflammatory events. Insulin and glucocorticoids, however, exert direct regulatory effects at concentrations usually found in plasma. Insulin is endowed with facilitatory actions on vascular reactivity to inflammatory mediators and inflammatory cell functions. Increased concentrations of circulating glucocorticoids at the early stages of inflammation results in downregulation of inflammatory responses. Oestrogens markedly reduce the response to injury in a variety of experimental models. Glucagon and thyroid hormones exert indirect anti-inflammatory effects mediated by the activity of the adrenal cortex. Accordingly, inflammation is not only merely a local response, but a hormone-controlled process.


Mediators of Inflammation, 2, 181–198 (1993)

### Hormonal control of inflammatory responses

J. Garcia-Leme[CA] and Sandra P. Farsky

Department of Pharmacology, Institute of Biomedical Sciences, University of São Paulo, 05508-900 São Paulo, SP, Brazil

[CA] Corresponding Author

**ALMOST any stage of inflammatory and immunological responses is affected by hormone actions. This provides the basis for the suggestion that hormones act as modulators of the host reaction against trauma and infection. Specific hormone receptors are detected in the reactive structures in inflamed areas and binding of hormone molecules to such receptors results in the generation of signals that influence cell functions relevant for the development of inflammatory responses. Diversity of hormonal functions accounts for recognized pro- and anti-inflammatory effects exerted by these substances. Most hormone systems are capable of influencing inflammatory events. Insulin and glucocorticoids, however, exert direct regulatory effects at concentrations usually found in plasma. Insulin is endowed with facilitatory actions on vascular reactivity to inflammatory mediators and inflammatory cell functions. Increased concentrations of circulating glucocorticoids at the early stages of inflammation results in downregulation of inflammatory responses. Oestrogens markedly reduce the response to injury in a variety of experimental models. Glucagon and thyroid hormones exert indirect anti-inflammatory effects mediated by the activity of the adrenal cortex. Accordingly, inflammation is not only merely a local response, but a hormone-controlled process.**

**Key words:** Glucagon, Glucocorticoids, Hormonal control of inflammation, Inflammation, Insulin, Oestrogens, Thyroid hormones

### Introduction

An impressive challenge imposed by external factors to an organism in the animal kingdom, particularly in vertebrates, is the reaction to emergency conditions, such as trauma and infection. To deal efficiently with the corresponding demands, the organism activates a narrowly regulated set of events, characteristic of the inflammatory response. These include functional alterations of microvessels, migration of haematogenous cells to the injured area, phagocytosis by competent cells and local pain. Recruitment of components of the immune system leads to the amplification of the reaction.

Inflammation was considered a disease until Hunter in 1794, described it as a nonspecific response evoked by all manner of injury and both purposeful and beneficial in nature. This view was strongly supported by the results of Metchnikoff's investigations on the comparative pathology of inflammation dating from the end of the nineteenth century. The beneficial character of the response, however, was not easily assimilated at the time, inflammation being rather regarded as a passive and deleterious reaction, of no benefit to the host. Experiments in which pyogenic bacteria were used as the inflammatory stimulus, convincingly illustrate the defensive aspect of inflammation. In these experiments, recognized components of the early inflammatory reaction were blocked by systemic shock, local ischaemia, or administration of anticomplementary substances, and the effect of a modified host resistance on the final size of the developing bacterial lesion was compared to the lesion size in control animals. In all cases, the injection of bacteria into the dermis during periods of shock or ischaemia, or following the administration of anticomplementary substances resulted in maximal lesions substantially larger and more severe than the control lesions.[1–4] The results indicate that there is an early period in which the integrity of the response against injury is essential to prevent or limit the intensity of a future lesion, and stress the defensive role of inflammation.

A most significant aspect in earlier views on inflammation is the notion that the response is a pattern reaction developing regardless of the cause. Accordingly, any type of injury tends to evoke a similar response of the organism. It was from such notion that the idea of the response being mediated by endogenously mobilized active materials—the inflammatory mediators—arose. Ideally, a potential inflammatory mediator should have the appropriate

© 1993 Rapid Communications of Oxford Ltd properties to bring about that part of the inflammatory response for which it is deemed to be responsible; it should be demonstrably present for a phase of the inflammatory response; and if it is possible to deplete the tissues of the mediator, this should also lead to a suppression of the inflammatory event for which it is responsible.[5,6]

From numerous observations, generally it has been concluded that, in addition to chemical mediators, the inflammatory response is under the influence of substances of diverse origin which are capable of modulating its development. It is possible to identify local tissue factors, plasma factors, neurogenic factors, and hormones which act as modulators of the inflammatory process.[7–10] These substances provide the basis for the occurrence of regulatory mechanisms responsible for a fine-tune adjustment of the host response in accordance with the intensity of the damage inflicted. Were it only merely a local response, inflammation would hardly be so effective as a defensive reaction against injury.

Hormone studies from the early decades in this century, clearly indicate that many biological systems are subject to control by hormone actions. Binding of hormone molecules to specific receptors in target structures results in the generation of signals that influence cell functions. Specific hormone receptors are detected in the reactive structures in inflamed areas and, in most instances, there is a striking similarity of the binding sites in those structures and those present in recognized target cells as far as the apparent affinity, specificity, and kinetics of the interaction are concerned. Accordingly, hormones can influence cell functions relevant for the development of inflammatory responses, including vascular reactivity to inflammatory mediators, oriented cell locomotion, particle ingestion by phagocytes, and microbicidal function. Granulation tissue formation, healing, and repair are also affected by the activity of endocrine glands. Diversity of hormonal functions is likely to account for the recognized pro- and anti-inflammatory effects exerted by these substances.

Most hormone systems are capable of influencing the development of inflammatory reactions.[10] Insulin and glucocorticoids, however, appear to exert direct regulatory effects on the reactive structures in an inflamed area, at concentrations usually found in plasma.

### The proinflammatory effect of insulin

*The insulin receptor:* Insulin occurs throughout the entire vertebrate kingdom and is probably also present in many invertebrates. It serves the essential role of regulating the synthetic phase of energy metabolism by promoting glucose and amino acid uptake, glycogen and protein synthesis, and lipogenesis. The existence of a specific insulin receptor was postulated as early as 20 years ago.[11–13] The receptor is a heterotetrameric structure consisting of two α-subunits of $M_r$ 135 kDa on the outside of the plasma membrane connected by disulphide bonds to β-subunits of $M_r$ 95 kDa which are transmembrane proteins. Insulin binding to the α-subunit induces conformational changes which are transduced to the β-subunit. This leads to the activation of a tyrosine kinase activity which is intrinsic to the cytoplasmic domain of the β-subunit. Activation of the tyrosine kinase activity of the insulin receptor is an essential step in the transduction of an insulin signal across the plasma membrane of target cells. Signal transduction on the postkinase level possibly involves phosphorylation of substrate proteins at tyrosine residues, activation of serine kinases, and interaction with G-proteins, phospholipases and phosphatidylinositol kinases.[14] A large number of agents and altered physiological conditions are known to affect insulin binding to target cells. Addition of catecholamines, glucagon, or glucocorticoids to cells, as well as the diabetic state and obesity modify insulin receptor activity.[15–18]

*Insulin and the endothelial cell:* Insulin receptors are demonstrable on the surface of endothelial cells.[19] Regional differences, however, are observed in insulin receptor concentration, as revealed by insulin binding studies in primary cultures of endothelial cells. Both arterial and venous endothelial cells possess typical receptors for insulin on the basis of specifity of binding, curvilinear Scatchard plots, affinity profiles, pH dependence, and dissociation kinetics. Arterial cells, however, bind at least 2.5 times more insulin than do venous cells, whether studied at 4, 24, or 72 h after *in vitro* plating.[20] Scanning electron microscopy studies indicate that overt differences in surface morphology and surface area do not account for the differences in insulin binding between the two cell types. The cause of the marked difference in binding is not readily apparent. The observation that endothelial cells from several vascular locations can also manifest differences in a diverse group of functional properties, however, suggests that endothelial cells, from different vascular sites possess highly specialized and distinct surface characteristics and functional properties.[20]

The characterization of insulin uptake by microvascular tissues is usually hampered by a lack of suitable tissue preparations and by the complexity involved in analysing binding studies in a tissue with more than one type of cell. Nevertheless binding of insulin to microvessels isolated from adult bovine and neonatal porcine cerebral cortex is shown to be affected by pH, temperature, and other physiological conditions in much the same manner as the binding of this hormone to liver, adipose tissue, and muscle. Furthermore, chemical covalent cross-linking of insulin to its receptor in cerebral microvessels and the subsequent isolation of the hormone–receptor complex, indicate that the hormone is found associated with a polypeptide with a molecular weight which is indistinguishable from the α-subunit of the liver insulin receptor.[21]

The presence of endothelial surface receptors for insulin may be of physiological significance in several general ways: insulin could directly modulate endothelial function; surface receptors for insulin could play a role in the transfer of insulin between the circulation and the subendothelial tissues; insulin bound to the endothelial surface could be released into the circulation, the endothelium serving as an additional storage compartment that would influence blood levels of insulin.[20] Therefore, though the mechanism of glucose entry into vascular endothelial cells is thought to be independent of the action of insulin,[22] the hormone is likely to influence the activity and functions of the vascular endothelium.[23,24]

*Vascular responsiveness in insulin-deficient states:* Diabetes mellitus, as a clinical or experimental entity, affects the microcirculation, as well as large arteries and occasionally veins. The ability of insulin to restore altered vascular responsiveness in experimental diabetes mellitus is an indication that the alterations are a consequence of the diabetic state. This is observed not only in preparations of large vessels from diabetic animals receiving insulin,[25,26] but also in microvascular beds of insulin-treated diabetic animals.

Data obtained with tyramine, an indirectly acting sympathomimetic amine, provide evidence that no abnormal release of noradrenaline from storage sites occurs at the early stages of diabetes mellitus. Furthermore, they indicate that uptake mechanisms into the adrenergic nerve terminal are unlikely to be altered since displacement of noradrenaline requires tyramine uptake. Interaction of noradrenaline with specific receptors to produce sympathomimetic effects is also unaffected as shown by equivalent arteriolar constrictor responses induced by the catecolamine in normal and diabetic animals. In addition, equal concentrations of phentolamine are capable of blocking such responses in both groups of animals. Dilatation of arterioles and venules is observed in normal and diabetic animals with equivalent doses of acetylcholine, thereby suggesting that effector mechanisms for vasodilation are also preserved at the early stages of diabetes mellitus. Accordingly, release of endothelium-derived relaxing factor (EDRF) by acetycholine does not appear to be altered, nor does the relaxing mechanisms in vascular smooth muscle, as shown by the responses to papaverine, an endothelium-independent vasodilator.[27] Therefore, if functional alterations of microvessels are observed at the early stages of the diabetic state, they are not necessarily related to any noticeable dysfunction of the autonomic nervous system. However, 2 months after the induction of streptozocin diabetes in rats, a nonspecific decrease in contractile responsiveness of the mesenteric vascular bed to nerve stimulation, and to intraluminal noradrenaline can be shown. The diabetes-evoked depression of responses at this stage suggests the occurrence of functional changes in vascular reactivity to which an abnormality in sympathetic neuronal activity contributes.[28]

Norepinephrine evokes a constrictor response of mesenteric microvessels *in situ*, the latency of which is analogous in normal and alloxan diabetic rats. Histamine and bradykinin are capable of antagonizing this response in normal but not in diabetic animals, unless the minimum effective doses are increased about 20-fold. In contrast, acetylcholine and papaverine are equally effective in both groups of animals. The altered response to histamine and bradykinin are not directly associated with hyperglycaemia since fasting renders the diabetic animals normoglycaemic and yet does not restore the reactivity of microvessels to these agents. Previous administration of insulin to diabetic animals corrects the impaired responses.

The functional changes observed in the responses to histamine and bradykinin, under these conditions, are unlikely to be associated with a defective response of the smooth muscle. First, because in extravascular smooth muscles obtained from either normal or diabetic animals equivalent responses to histamine and bradykinin are observed. Secondly, because concentration–effect curves, constructed from the response of isolated aortae to noradrenaline, are similar in normal and diabetic animals, provided the endothelium is removed. Differences are only observed in preparations in which the endothelium is left intact. Since histamine and bradykinin are potent permeability-increasing agents in most species, whereas acetylcholine and papaverine are devoid of such an action, histamine and bradykinin might antagonize the constrictor response of microvessels to noradrenaline through an action on lining endothelial cells resulting in interendothelial gaps and increased vascular permeability with transitory changes in the composition of extravascular fluid.[29,30] Induced changes in local steady-state conditions, if the only from the osmotic point of view, can affect the reactivity of small vessels.[31] Accordingly, endothelial cells may play a role when vasoactive substances, endowed with permeability-increasing properties, act on microvessels. That insulin is involved in such events is suggested by the restorative effect it exerts in diabetic animals and by the finding that a similar condition of impaired responses to histamine and bradykinin is produced in normal animals by the injection of 2-deoxyglucose, the acute effects of which are the result of intracellular glucopenia secondary to inhibition of glucose utilization.[32]

The findings are consistent with the view that functional alterations of microvessels, characteristic of the diabetic state, involve the vascular endothelium and that, at least in part, they may be linked to continuing insulin deficiency.

*Insulin and the vascular response to injury:* The observations described above point to the involvement of insulin in events that are important for the development of inflammatory responses. It is, therefore, expected that many aspects of inflammation will be affected by the diabetic state. Edlung and co-workers in 1952 reported in a brief note that alloxan inhibits the dextran oedema in the rat. The effect, however, was attributed to alloxan itself and not to the diabetic state.[33] Subsequent studies revealed that regardless of what the immediate effects of alloxan may be, decreased inflammatory reactions in alloxan diabetic animals are obviously related to the diabetic state, since they can be obtained for several days following the injection of alloxan and can be reversed by insulin treatment. Alloxan diabetic rats fail to present the characteristic cutaneous oedema ('anaphylactoid' response) which follows the intravenous injection of dextran or egg white. The histamine content of the skin in these animals, however, is indistinguishable from that of controls. Pretreatment with insulin restores the ability of the animals to respond in a normal fashion to the injection of dextran or egg white. Furthermore, compound 48/80, a potent histamine releasing agent, is capable of evoking a shock-like picture of comparable intensity in alloxan treated and normal animals, thus indicating that the reduced inflammatory response rather reflects a reduced capacity of the animals to respond to inflammatory agents.[34] In addition, insulin considerably sensitizes rats to the dextran anaphylactoid reaction. Ineffective doses of dextran can initiate the reaction if given in association with crystalline insulin.[35]

Light microscope investigation and electron microscopic studies revealed that microvessels of alloxan diabetic rats, challenged with histamine or serotonin, exhibit less labelling by intravenously injected colloidal carbon particles than do vessels of normal animals. Insulin corrects this condition and also potentiates leakage of carbon evoked by permeability-increasing factors in normal animals. The carbon particles label venules almost exclusively and abandon the lumen of leaking vessels through interendothelial junctions.[36] The vascular endothelium is highly permeable to water and water soluble molecules, but normally restricts the passage of macromolecules. Escape of plasma proteins in inflammation (or of colloidal carbon particles as in the experiments referred to above) apparently depend on a partial 'disconnection' of endothelial cells along the intercellular junctions. Several mechanisms were proposed in the attempt to explain how the molecules of inflammatory mediators succeed in creating intercellular gaps in microvessels through which leakage of macromolecules occurs. Contraction of endothelial cells is still a tenable explanation.[37–41] Insulin through an action on endothelial cells, might influence the response of these cells to inflammatory mediators, thus exerting a facilitatory effect on microvascular permeability.

This view is supported by the following observations. The oedema which develops in an area after local injection of chemical irritants or application of physical stimuli is reduced when the animals are rendered diabetic by the administration of alloxan or streptozocin or by subtotal pancreatectomy. This inhibition is abolished by previous injection of insulin and is not associated with increased blood sugar concentrations. If diabetic animals are fasted for a few hours with water *ad libitum*, normal glucose levels are regained, but the animals still present reduced responses to the irritants. Accordingly, insulin deficiency, more than hyperglycaemia, is likely to be the cause of oedema inhibition in diabetic animals.[42,43] Diabetes mellitus does not affect the endogenous release of vasoactive agents, as referred to above. Furthermore, equivalent *in vitro* release of histamine from mast cells or of kinins from plasma by specific releasing agents is observed irrespective of whether the cells or plasma are obtained from normal or diabetic animals. In addition, diabetic animals exhibit decreased responses, in comparison with controls, to permeability factors such as histamine, bradykinin, or serotonin injected into the skin. Therefore, even if an irritant is capable of releasing permeability factors in diabetic animals, the ultimate effect of such factors on the microvasculature of the affected area is decreased, thus suggesting a modulatory role for insulin at this level.[42,43] Reduced responses to intracutaneous permeability factors are also observed in normal animals previously given 2-deoxyglucose to inhibit glucose utilization. Insulin does not improve the response of 2-deoxyglucose treated animals to the permeability-increasing agents.[32] The fact appears to indicate that whenever glucose utilization by the reacting structures is impaired, reduced microvascular permeability responses can be observed. The activity of vascular endothelial cells may, therefore, be metabolically regulated by insulin.

Activation of sensory neurons in unmyelinated fibres results in arteriolar dilatation and plasma exudation. The response is known as neurogenic inflammation and presumably involves the release of substance P and related substances. Neurogenic inflammation is also reduced in alloxan diabetic rats.[43] Furthermore, a marked decrease in plasma leakage evoked by substance P is observed in rats rendered diabetic by the administration of streptozocin. Determination of the content of substance P in sensory nerves and spinal ganglia, however, shows that it is not altered by the diabetic state.[44]

In contrast with the observations described above, altered transendothelial changes with diffusion of serum proteins to perivascular tissues, particularly at the capillary level of the microcirculation, may accompany the progression of the diabetic state. These changes, however, are not 'inflammatory' in their nature but rather reflect the occurrence of metabolic disorders responsible for the development of the diabetic microangiopathy. Recent evidence indicates that the endothelial cell, via specific receptors, is capable of responding to nonenzymatic glycosylation products originating from the interaction of simple sugars with amino groups. Nonenzymatic glycosylation is enhanced in patients with diabetes mellitus. When glycosylation-modified albumin is added to cultured endothelial cells, an increased permeability is observed. Accordingly, extravasation of serum proteins in diabetic vascular disease, unrelated to inflammatory stimuli, at least in part, appears to depend on the response of endothelial cells to glycosylation end products.[45]

*Insulin receptors in inflammatory cells:* In white cells, the insulin binding can be studied under conditions that are most nearly physiological, employing normal cells or cells obtained from patients with clinical disorders. Furthermore, cultured cells can also be used. Granulocytes, mononuclear leukocytes, granulocytic leukaemic white cells, cultured human lymphocytes—and human erythrocytes and cultured fibroblasts as well—all have specific insulin binding sites. These cells bind $^{125}$I-insulin, and the bound hormone is displaced by nanogram quantities of unlabelled peptides.[46–50] When freshly isolated circulating leukocytes (monocytes) are incubated with $^{125}$I-insulin and examined by electron microscopic autoradiography, about 20% of the labelled material is internalized after 15 min at 37°C. By 2 h at the same temperature, approximately one-half of the $^{125}$I-insulin is internalized. The leukocytes bind and internalize insulin in a manner that mirrors that of major target tissues, such as the hepatocytes.[51] Since cell surface receptors are internalized with the ligand, this provides a mechanism to initiate cell surface receptor loss. After removal of insulin from the incubation medium, the internalized receptors are rapidly reinserted back into the cell surface.[52] Accordingly, the concentration of available insulin receptors on the cell surface and, consequently, the sensitivity of the cell to insulin, are regulated by the ambient concentration of insulin.

Cultured macrophages and macrophage-containing cell populations both possess specific receptors for insulin with properties indistinguishable from those of human leukocytes and other mammalian tissues.[53] In addition, platelets contain specific receptors for insulin.[54] Therefore it is entirely possible that a variety of functions of circulating leukocytes and platelets and of tissue macrophages are under the influence of insulin.

Studies on polymorphonuclear leukocytes obtained from diabetic patients greatly enhanced the knowledge of the role played by insulin in metabolic functions of inflammatory cells. The available evidence points towards glucose being freely permeable to the cell membrane. Comparative investigations show that the glycolysis of diabetic cells is decreased relative to controls. Glucose-6-phosphate and fructose-6-phosphate accumulate in diabetes, suggesting a decreased activity of phospho-fructokinase. The increase in glucose-6-phosphate concentration is probably responsible for the decrease in glucose utilization. The amount of glycogen and its synthesis is markedly reduced in diabetes. Insulin *in vivo* corrects the metabolic alterations observed in polymorphonuclear leukocytes.[55]

*Abnormalities of leukocyte functions in diabetes mellitus:* An enhanced susceptibility to infection is thought to occur in a poorly controlled diabetic state. In addition, critical evaluations of the topic show that infection is more serious and possibly more difficult to eradicate in the diabetic host.[56,57] This might reflect an altered capacity of the diabetic patient to mount an inflammatory response, and the occurrence of immunoregulatory disorders.

Granulocyte dysfunction is a common finding in diabetes mellitus. Investigations in well-controlled diabetic subjects may fail to demonstrate any consistent defect that might predispose the patient to infection. However, in poorly controlled diabetic states, and in experimental diabetes mellitus, abnormalities in granulocyte chemotaxis, phagocytosis and microbicidal mechanisms are described.

Disturbances of the inflammatory cycle evoked by diabetes mellitus with reduction of leukocyte migration to the inflamed lesion are not uncommon. They have been clinically recognized.[58] For instance, the early granulocyte phase of the local cellular response in surgically abraded lesions is significantly delayed and diminished in poorly controlled diabetic patients.[59] Furthermore, the mean chemotactic index in diabetic patients is significantly less than in matching controls. The defect in chemotaxis is not correlated with plasma glucose levels, serum carbon dioxide, and blood urea nitrogen values.[60–63]

The early local exudative cellular reaction in an inflammatory lesion is impaired in alloxan-induced diabetic rats due to a reduced migration of neutrophils to the inflamed area. Neutrophils, however, are capable of moving from reserve compartments (inflammatory leukocytosis) into blood in these animals, as much as in controls. Furthermore, an intrinsic cellular defect does not occur, because leukocytes obtained from diabetic animals are not devoid of chemotactic responsiveness when suspended in culture medium or normal serum. Suspended in the corresponding 'diabetic' serum, blockade of chemotaxis to lipopolysaccharide (LPS)-activated serum is observed. Hyperglycaemia alone, or hyperosmolality secondary to hyperglycaemia, the presence of ketone bodies, malnutrition, or a direct effect of alloxan do not explain the results. In addition, the capacity to generate chemotactic agents remains intact in serum of diabetic animals. The defect appears to be due to the presence of an inhibitory factor in plasma which is heat labile (56°C), is destroyed by incubation with trypsin, and is retained after dialysis with 10 000 $M_r$ retention dialysis tubing. Pretreatment of the animals with insulin results in the recovery of the chemotactic response either *in vitro* or *in vivo*.[64] The inhibitory activity of chemotaxis is detected shortly after the onset of the diabetic state, being observed from the third day of alloxan administration.[65] The neutrophil migratory response *in vitro* to the chemotactic peptide *N*-formyl-methionyl-leucyl-phenyl-alanine (fMLP), and to leukotriene (LT) $B_4$ is not affected by the presence of diabetic rat serum.[66] Bacterial lipopolysaccharides have been demonstrated to activate both the classical and the alternative pathways of complement by a mechanism that does not require antibody to the LPS molecules. This results in the rapid appearance of chemotactic activity. Distinct subsets of chemotactic receptors mediate the response of neutrophils to fMLP, $LTB_4$ and complement-derived chemotaxins. Accordingly, the inhibitory activity of chemotaxis developing in diabetic rat serum appears to be restricted to some stage of the interactive process between neutrophils and complement-derived chemoattractants.

The mechanisms underlying leukocyte accumulation in a tissue depend on the interaction between the cells and the vascular endothelium. The contact between white cells and the vessel wall, particularly in venules, may take the form of either rolling of leukocytes along the endothelium or their stationary adhesion. If adhesive forces are sufficient to fully counter the shearing forces exerted by the blood stream, then stationary leukocyte adhesion will result. In the case of lesser adhesive forces, leukocyte rolling may occur, provided the shearing forces remain below a critical level.[67] During inflammation leukocyte–endothelial interactions progress to a point where adherence and emigration of the cells occur. Defective leukocyte–endothelial interactions are observed *in situ* in diabetic states. Under basal conditions, the number of rolling cells in postcapillary venules of transilluminated tissues of diabetic rats is markedly reduced relative to controls. The finding is not dependent on the number of circulating leukocytes because total and differential counts in the peripheral blood are equivalent in both groups. If a noxious stimulus is applied to induce a local lesion, leukocytes accumulate in the connective tissue of normal animals in a pattern characteristic of the inflammatory reaction, whereas in diabetic rats only a few cells are found in an equivalent area of the perivascular tissue. Reversal of the defective leukocyte–endothelial interaction is attained by treatment of diabetic animals with insulin. Control rats injected intravenously with lyophilized plasma constituents, obtained after dialysis of diabetic rat plasma with 12 000 $M_r$ retention dialysis tubing, behave as diabetic animals in that they exhibit a reduced number of leukocytes rolling along the venular endothelium. Heating of active samples for 1 h at 56°C results in the complete loss of the inhibitory effect.[68]

The findings indicate that substances present in plasma of diabetic subjects are capable of influencing leukocyte–endothelium interactions and chemotaxis, thereby reducing the number of cells which accumulate in an inflamed area. What is still incompletely defined is the precise nature and origin of these substances. Several families of adhesion receptors which play a role in leukocyte–endothelial interactions and, possibly, chemotaxis have been identified. These include: the integrins; the adhesion molecules of the immunoglobulin superfamily; and cell adhesion molecules with lectin-like domains.[69] Disturbances of the inflammatory cycle evoked by diabetes mellitus—and characterized by a reduced leukocyte migration to the inflamed area—might ultimately reflect an interference of plasma factors on the activity of such receptors.

Much effort has gone into studies of the phagocytic and microbicidal function in diabetes mellitus and, with a few exceptions, results reveal impairment of these functions. The percentage of neutrophils actively engaged in phagocytosis is considerably less in diabetic rats and the average number of organisms which have undergone phagocytosis by these cells is reduced relative to cells of normal control animals. However, the differential cell counts of the peripheral blood of the alloxan diabetic rats are equivalent to those of controls.[70] In addition, alloxan diabetic rats are more susceptible to experimental pneumococcal pneumonia than nondiabetic rats. The cumulative mortality in the diabetic group is significantly higher and more than ten times as many viable pneumococci are found in the pneumonic lesions of these animals as are present in the lesions of the nondiabetic controls. Serial histological studies revealed that phagocytosis is strikingly depressed in the alveolar exudates of the diabetic animals.[71]

To separate ingestion from killing in 'diabetic' granulocytes, a technique was developed which distinguishes whether the altered efficiency of microbial killing is a consequence of an impairment in intracellular mechanisms or simply a consequence of a slower rate of microbial ingestion. From these studies it was concluded that diabetic subjects have a neutrophil phagocytic defect, an impaired intracellular killing, or a combined phagocytic and intracellular killing defect.[72] Since a clear-cut improvement in the microbicidal function of granulocytes is achieved following intensive diabetes management and reduction of fasting glucose concentrations, insulin may also participate in metabolic pathways regulating intracellular microbial killing. Glucose is freely permeable to the polymorphonuclear membrane. Most glucose, however, is metabolized via the glycolytic pathway and some enzyme reactions in this pathway are closed regulated by insulin availability. This suggests that the impairment in microbicidal functions in diabetes mellitus may have a metabolic basis.[73]

Diabetes mellitus may also affect the mononuclear phagocytic system. Macrophages obtained from the peritoneal cavity of alloxan diabetic rats exhibit a reduced capacity to engulf opsonized sheep erythrocytes when compared with the activity of cells obtained from matching controls. Recovery of the impaired response is attained by pretreatment of the animals with insulin.[65]

*Immune responses:* With the use of monoclonal antibodies to characterize lymphocyte subsets it was suggested that patients with type-I (insulin-dependent) diabetes mellitus may present immunoregulatory disorders already at the onset of the disease due to quantitative imbalance of lymphocyte subsets. In type-I diabetes of long standing the total T-cell population is reduced. Type-II (noninsulin-dependent) diabetic patients, however, show no abnormalities in T-lymphocyte subsets.[74] Suppressor T-cell activity is reportedly defective when lymphocytes from insulin-dependent diabetic patients are stimulated by concanavalin A. The abnormality may be related to a paucity of suppressor T-lymphocytes as evaluated by specific monoclonal antibodies.[75–78] Cell-mediated immune responses are significantly reduced in human insulin-dependent diabetes,[79] as well as the production of IL-2.[80,81]

Comparison of antibody responses in normal and diabetic animals has not always conclusively revealed a significant difference between both groups.[82] Diabetic and control rats sensitized by the subcutaneous injection of a solution of ovalbumin exhibit equivalent IgE antibody titres, as measured by homologous passive cutaneous anaphylaxis. However, a marked reduction in the number of leukocytes present in the bronchoalveolar lavage fluid after antigen challenge is observed in diabetic animals. Pretreatment with insulin restores the response to normal levels. The finding implies that despite equivalent antibody production, sensitized diabetic animals are unable to react normally to antigen challenge, and that the impaired response is likely to depend on the continuing deficiency of insulin associated with the diabetic state.[83]

*Conclusions:* Overall, the experimental findings commented upon in the preceding sections agree with

**Table 1.** Evidence supporting the proinflammatory effect of insulin

| | Reference no. |
|---|---|
| Occurrence of specific receptors on the surface of endothelial and inflammatory cells. | 19–21,46–53 |
| Quantitative alterations of inflammatory events in insulin-deficient states: | |
| (a) Decreased microvascular responses to inflammatory mediators; | 29,30,32,34,35 |
| (b) Reduced microvascular leakage and oedema formation; | 36,42–44 |
| (c) Deficient leukocyte–endothelial interactions and reduced accumulation of cells in inflammatory exudates; | 59,68 |
| (d) Granulocyte dysfunctions characterized by chemotactic, phagocytic and intracellular killing defects; | 60–64,66,70–72 |
| (e) Impaired function of the mononuclear phagocytic system. | 65 |
| Restorative effect of insulin unrelated to the correction of hyperglycaemia and plasma osmolality (in insulin-deficient states). | 29,30,32,34,36, 42,43,64,65,68,83 |
| Enhanced susceptibility to infection in patients with diabetes mellitus; infections are more serious and possibly more difficult to eradicate. | 56,57 |

the clinical evidence that disturbances of the inflammatory cycle are not uncommon in the diabetic state, rendering the diabetic patient more susceptible to infections. Under normal conditions the activity of the pancreatic islet B cells supplies optimal concentrations of circulating insulin which is then immediately available to target structures. Insulin receptors can be identified in the reacting structures of an inflamed area. It is plausible, therefore, to assume that the activity of those structures is under control by the ambient concentration of the hormone. Altered vascular responsiveness, defective leukocyte–endothelial interactions, and inflammatory cell dysfunctions resulting in chemotactic, phagocytic, and intracellular microbial killing defects are described in insulin-deficient states. In most instances, pretreatment with insulin corrects the alterations observed, thereby indicating that a continuing deficiency of the hormone is a major element in conditioning impaired responses to noxious stimuli. Evidence for the proinflammatory role of insulin is summarized in Table 1.

### Glucocorticoids and the feedback mechanism regulating the progression of inflammatory responses

*The glucocorticoid receptor:* The molecular mechanisms by which glucocorticoid effects are produced have many common features with those of other classes of steroids. They readily penetrate the cell membrane. In the cell the hormone binds to receptor proteins and exerts its effects through receptor-mediated genomic actions. From numerous observations, generally it has been concluded that the uncomplexed glucocorticoid receptor is a heteromer composed of a single steroid and DNA-binding subunit, and two 90 kDa heat shock proteins (hsp90). Such proteins are abundant cytoplasmic materials. The DNA-binding domain is a highly conserved sequence of about 65 amino acids required for interactions with the glucocorticoid response elements (GREs), that correspond to short sequences of DNA to which the receptor binds to alter transcription. The carboxy-terminal amino acids comprise the steroid binding domain that, in the absence of the ligand, appears to maintain the receptor in a repressed state. Binding of the hormone to its receptor results in the formation of a complex that undergoes activation with acquisition of an enhanced affinity for DNA. Activation is thought to involve a conformational change of the hormone–receptor complex with dissociation of the hsp90 subunits. Translocation of the activated complex into the cell nucleus forms the nuclear bound complex, primary effects of glucocorticoids being exerted at the gene level and resulting in transcriptional induction or repression. In humans, the glucocorticoid receptor is found in nearly all cell types. The molecular basis for target cell specificity cannot, therefore, be due to cell-specific expression of the receptor, but is more likely controlled at the level of the target gene itself.[84–90]

*Glucocorticoid administration and the sequence of inflammatory events:* Glucocorticoids have long been used for the management of inflammatory diseases and deleterious immune responses. Steroids employed in glucocorticoid therapy are usually analogues of cortisol, the major active glucocorticoid in humans. Therefore, their administration under experimental conditions greatly helps the understanding of glucocorticoid action.

*The microcirculation:* Glucocorticoids are known to play a role in the maintenance of the functional integrity of the microcirculation. Corticosterone depresses the permeability effects of inflammatory mediators in normal animals, while the enhanced permeability response in adrenalectomized animals is decreased by the steroid to levels somewhat lower than in controls.[91] Dexamethasone significantly decreases permeability reactions caused by compound 48/80, calcium ionophore, hypotonic salt solutions, histamine, serotonin, platelet-activing factor (PAF), bradykinin and $LTC_4$ and $LTD_4$. Maximum inhibitory effects are obtained a few hours after drug administration.[92] Passive cutaneous anaphylaxis evoked in mice is blocked by glucocorticoids. A latency period is required for the expression of the inhibitory effect.[92] In addition, vascular leakage in an inflammatory reaction induced by immune complexes is counteracted by the steroids.[93] In exteriorized tissues, local exposure of the microcirculation to a glucocorticoid blocks microvascular leakage evoked by histamine or $LTB_4$. The effect, observed after a latency period, apparently is exerted at the vascular endothelial cell level.[94,95] Glucocorticoid receptors are demonstrable in cultured endothelial cells and constitute a population of high-affinity sites in these cells,[96] where the steroids exert several effects including changes in morphology and protein synthesis.[97]

*Accumulation of leukocytes in inflammatory infiltrates:* Leukocytes always adhere to the vascular endothelium before penetrating the vessel wall. Two categories of substances formed or released in an inflamed area enhance leukocyte adherence to endothelial cells: chemotactic products and cytokines. The effect of chemoattractants (complement-derived factors, fMLP, $LTB_4$) is primarily directed towards the leukocyte, since pretreatment of the endothelial cell with such substances usually fails to enhance adherence. In addition, cytokines such as tumour necrosis factor (TNF) and granulocyte-macrophage colony-stimulating factor (GM-CSF) also enhance the ability of neutrophils to adhere to the endothelium.[98–102] The stimulation is usually transient. Endothelial cells *in vitro* are stimulated by TNF and interleukins (IL) to become more adhesive to neutrophils.[99–103] As referred to above, specific adhesion glycoproteins expressed on the surface of leukocytes and endothelial cells play a relevant role in the adhesive phenomenon. Chemotactic factors acting on leukocytes and cytokines acting on both the leukocyte and the endothelial cell induce the expression of adhesion molecules on their surface favouring cell-to-cell interactions. IL-1 is produced by nearly all cell types in response to antigens, toxins, and inflammatory processes. It is capable of inducing synthesis of other cytokines (lymphokines) and shares many of its properties with TNF.[104,105] Reduction of serum IL-1 activity is observed after treatment with glucocorticoids.[106] Glucocorticoids decrease the production of IL-1 by macrophages.[107] Treatment of human lung fragments *in vitro* with glucocorticoids results in a dose-dependent inhibition of IL-1 production. Nonglucocorticoid steroids have no effect.[108] Glucocorticoids strongly inhibit TNF production. The inhibiting effect may be demonstrated *in vitro* in the presence of cortisol concentrations corresponding to normal free cortisol levels *in vivo*.[105] Since locomotion of leukocytes in response to a chemical gradient of chemotactic factors is thought to remain unaffected following exposure of the cells to glucocorticoids,[109] the steroids, at least in part, appear to inhibit leukocyte accumulation in an inflamed area by blocking the production of mediators, particularly cytokines, involved in the recruitment process. The steroids might also inhibit the expression of adhesive molecules on the surface of inflammatory and endothelial cells.

Both hydrocortisone and betamethasone have a suppressive effect on neutrophil mobilization into the peritoneal cavity of mice and rats injected with living microorganisms, endotoxins, or bacterial extracts. Betamethasone also decreases by more than 50% the number of neutrophils which are mobilized into the pleural cavity of rats injected with kaolin. Several anti-inflammatory steroids are capable of reducing leukocyte emigration into the pouch fluid when tested by suspending them directly in the irritant solution employed to produce the response. Labelled leukocytes previously incubated with hydrocortisone and transferred to untreated recipients fail to accumulate at the inflamed site.[110,111]

*Phagocytosis and microbicidal activity:* In many instances, the effects of glucocorticoids on phagocytosis and microbicidal activity are dependent on the concentrations used. *In vitro*, high concentrations of the steroids are required to block phagocytic functions and microbicidal capability of polymorphonuclear leukocytes. Moderate to severe impairment of neutrophil phagocytosis occurs when cortisone is administered daily to human subjects for 7–9 days. In addition, lysosomal membrane stabilization as measured by the retarded release of acid phosphatase, and impairment in aerobic lactate formation and glucose utilization are observed with the use of cortisone. Hydrocortisone impairs the bacterial capacity of human polymorphonuclear leukocytes, and decreases $O_2$ consumption, the production of $H_2O_2$, glucose utilization, and the extracellular release of granular enzymes by the cells.[112,113]

In normal mice, hydrocortisone does not alter the number of macrophages already present in the peritoneal cavity, but the transit of mononuclear phagocytes from the circulation into the peritoneal cavity is arrested. However, during an inflammatory response in the peritoneal cavity, hydrocortisone is able to suppress the increase in number of peritoneal macrophages. This effect appears to be due to a diminished influx of mononuclear phagocytes from the peritoneal blood. Hydrocortisone, however, was found to have no effect on phagocytosis by mouse peritoneal macrophages.[112,113]

*Leukocyte kinetics:* Because glucocorticoids are capable of affecting the kinetics of circulating leukocytes, their adminstration causes marked changes in blood leukocyte counts. Administration of glucocorticoids leads to a decrease in the number of lymphocytes, monocytes, eosinophils, and basophils in the blood, whereas the number of neutrophils increases.

There are considerable differences in susceptibility to glucocorticoids among various species. Although the basis of these differences is not clearly understood, animal species have been divided into steroid-sensitive (hamster, mouse, rat, rabbit) and steroid-resistant (monkey, guinea-pig, humans) groups. The differentiation is usually based on the relative ease of producing lymphoid depletion after a given regimen of systemic glucocorticoids. In addition, there is a heterogeneity in the response of lymphoid cells to glucocorticoids even within the same species.[114]

In steroid-sensitive species lymphocytopenia results from a significant lympholytic effect of the glucocorticoids. Apparently, the lympholytic effect is a receptor-mediated event.[114] In resistant species, the decrease in number of circulating lymphocytes involves redistribution of the cells from the circulation into other body compartments. Even suprapharmacological concentrations of glucocorticoids are not capable of inducing lymphocyte lysis in humans.[114,115] Both T- and B-cells are depleted from the circulation, but there is a relative greater effect on the T-cell as compared to other lymphocyte subpopulations.[115,116] A transient monocytopenia with a return to normal counts within approximately 48 h, and eosinopenia occur in sensitive and resistant species following glucocorticoid administration. The response is apparently dependent on redistribution of the cells.[116] Steroid-induced neutrophilic leukocytosis reaches a peak 4–6 h after drug administration. The response is thought to occur mainly by an enhanced mobilization of cells from the marginated granulocyte pool.[112] Neutrophilia induced by glucocorticoids resembles peripheral blood changes produced by epinephrine and exercise. In these circumstances, the time to achieve peak responses is short, the neutrophil alkaline phosphatase content of cells is low which may signify re-entry or shifting of older mature cells from the marginated pool, and the number of immature cells in the peripheral blood remains low. Following administration of glucocorticoids the time to peak neutrophilia is also relatively short; the alkaline phosphatase content that is inversely related to neutrophil age is normally reduced in released cells; and immature neutrophils in the peripheral blood are practically absent. These findings corroborate the suggestion that development of neutrophilia following glucocorticoid administration is primarily due, at least in humans, to mobilization of the marginated granulocyte pool.[112]

*The immune system:* In many instances, undesirable consequences of an antigenic challenge can be controlled by the use of glucocorticoids. The control is chiefly the result of glucocorticoid effects on cytokine production or cytokine actions.

An antigen processed by the macrophage is presented to resting T-cells together with a major histocompatibility antigen. Under the influence of IL-1, secreted by the macrophage, the resting T-cell becomes activated and synthesizes a variety of proteins, of which some are released and others become integral components of the cell membrane. Gamma-interferon (γ-IFN), IL-2, -3 and -4, as well as the B-cell differentiating factor (BCDF) are released. Receptors for IL-2 and other cytokines are examples of membrane-associated proteins generated during antigenic challenge. The antigen can also activate B-cells through surface antibodies. B-cells under the influence of γIFN, IL-1, -2 and -4, and BCDF become antibody-secreting plasma cells. Activated T-cells undergo clonal expansion under the influence of IL-2, that also activates a particular group of cells to become cytotoxic lymphocytes. The cell-mediated response is amplified by γIFN, that enhances the processing of antigen by macrophages.[104]

Suppression of antibody production by glucocorticoids can be observed in some species,[114] but in humans, where blockade of immunological reactions by glucocorticoids has been described, it appears to have no accompanying effect on antibody synthesis.[114,116] Patients on glucocorticoid therapy exhibit a nearly normal antibody response to antigenic challenge.[117] Furthermore, there is no convincing evidence at present to suggest that glucocorticoids directly affect the complement system.[118] Glucocorticoid therapy does not significantly suppress the immediate wheal-and-flare response to an antigen skin test in atopic patients, thus suggesting that histamine release is not altered by the steroids.[118]

Monocytes and macrophages are among the most sensitive cells to the actions of glucocorticoids, whereas the lymphocyte is relatively resistant. Inhibition of recruitment of macrophages to an affected area is achieved by the use of glucocorticoids. Though production of macrophage migration inhibitory factor (MIF) by sensitized lymphocytes is not affected, the effects of MIF on macrophages are blocked by glucocorticoids.[116,119] Antigen uptake by macrophages is not influenced by the steroids.[119] However, the facilitatory effect of γIFN on the processing and display of antigens is inhibited. This suggests that activation of macrophages is regulated by opposing actions of cytokines and glucocorticoids. In addition, glucocorticoids decrease the production of IL-1 as referred to above. Two distinct forms of IL-1, termed IL-1α and IL-1β, have been purified and cloned. Production of both forms of IL-1 is suppressed by the steroids in a dose-dependent fashion and this is accompanied by equivalent reductions in mRNA expression for IL-1.[120] Accordingly, glucocorticoids through inhibition of IL-1 production are capable of influencing activation of resting T-cells. Furthermore, glucocorticoids reduce the generation of IL-2 and inhibit expression of IL-2 receptors on T-lymphocytes.[121–123] Therefore, T-cell proliferation is effectively blocked by the steroids, since IL-2 directs clonal expansion of specific T-lymphocytes. A direct consequence of these effects is the attenuation of cell-mediated immune responses.

*Endogenous glucocorticoids and the control of inflammatory responses:* Increased blood concentrations of glucocorticoids are observed after injection of endotoxins. This increase results from an enhanced release of steroids by the adrenal gland rather than from decreased metabolism of the normally secreted adrenal steroids.[124] Hypophysectomy abolishes the adrenal cortical responses to endotoxins, thereby implying that endotoxins do not act directly upon the adrenal cortex. Since blockade of the hypothalamic release of corticotropin-releasing hormone (CRH) also abolishes the increase of plasma glucocorticoids resulting from the injection of endotoxins, the substances appear to cause an increase of plasma corticosteroids by an action on the hypothalamus–pituitary–adrenal axis at the level of the nervous system.[125]

During the early stages of experimentally induced inflammatory responses, blood levels of glucocorticoids are consistently and markedly increased.[126–128] Evidence has been obtained that the development of acute inflammatory responses is regulated by a feedback mechanism which is induced comparatively early. In carrageenan or dextran lesions of the rat's paw, a factor has been harvested in perfusates of lesions 2–3 h in age, though not less than 1 h old. The effects of the factor are demonstrable by a decrease of exudation in other animals given the perfusate intravenously prior to injection of an irritant into the paw. Inhibition of oedema formation is abolished by prior adrenalectomy or electrolysis lesions in the hypothalamic median eminence of the test animals. The perfusate also elevates the serum level of corticosterone, concomitantly depressing the content of ascorbic acid of the adrenal. On the other hand, the perfusate is effective in rats whose adrenal medulla has been ablated, suggesting that adrenal catecholamines play no significant role in the inhibitory effect. Accordingly, the factor harvested in perfusates from inflamed lesions seems to owe its effects to the release of glucocorticoids (corticosterone) via the hypothalamus–pituitary–adrenal axis. In addition, when both hind paws of the rat are injected simultaneously with an irritant, responses of the same magnitude and parallel time-course developments are observed. If the injections, however, are made at an interval of 2.5 h, the paw that is first injected exhibits a normal response, whereas the response in the other paw is reduced to about 50%. This attenuation is not observed in adrenalectomized animals. These data have been interpreted to indicate that glucocorticoids secreted in larger amounts during the early stages of an inflammatory reaction, apparently govern the development of such reaction. The increased secretion, induced via the hypothalamus–pituitary–adrenal axis under the influence of factors produced in the inflamed area would thus represent the last event of a feedback mechanism that regulates the progression of inflammatory responses.[126] More recent investigations support the occurrence of a regulatory mechanism governing the development of inflammation. First, a relationship between the temporal development of carrageenan-induced inflammatory lesions and pulsed release in plasma of increased amounts of corticosterone in rats was reported.[128] Secondly, it was shown that susceptibility of inbred Lewis female rats to develop arthritis in response to streptococcal cell wall peptidoglycan polysaccharide (SCW) was related to a defective hypothalamus–pituitary–adrenal axis responsiveness to inflammatory mediators.[129] Thirdly, it was observed that resistance of histocompatible Fisher rats to SCW arthritis is regulated by an intact hypothalamus–pituitary–adrenal axis-immune system feedback loop.[129]

IL-1 provokes the secretion of adrenocorticotropic hormone (ACTH) and corticosterone in mice and rats.[130] Immunoneutralization of CRH blocks the stimulatory effect of IL-1 on glucocorticoid secretion, pointing to an activation of CRH secretion in response to the cytokine.[131] Similarly, IL-6 stimulates the secretion of ACTH in conscious, freely moving rats. The effect can be abolished by a previous injection of antiserum against CRH, thereby indicating that one site of action of IL-6 is at the hypothalamic level. Accordingly, a mutual regulatory control mechanism occurs between the reacting structures in an inflamed area and the activity of the hypothalamus–pituitary–adrenal axis.[132] The regulatory mechanism apparently involves the participation of cytokines generated at the inflamed site.

Inflammatory cell functions are also markedly influenced by the level of circulating glucocorticoids. The number of leukocytes rolling along the venular endothelium of the microcirculation network is markedly increased in adrenalectomized rats relative to sham-operated and normal controls. Blood leukocyte counts, however, are equivalent in the three groups of animals. No noticeable haemodynamic changes occur to explain the increase in number of rolling leukocytes in adrenalectomized rats. The rolling movement that represents the primary leukocyte–endothelial interaction, is presumably the result of two forces, one an adhesive force towards the wall of the vessel and the other the shearing force of the flowing blood. In a given venule with approximately constant blood flow velocity the shear force also remains constant.[133] Adhesion changes affecting the primary leukocyte–endothelial interaction are likely, therefore, to prevail following the ablation of the adrenal glands, resulting in an increased number of rolling leukocytes. That the effect is under control by glucocorticoids is indicated by the fact that methyrapone-treated animals also exhibit an enhanced number of cells rolling along the venular endothelium. Methyrapone, through inhibition of the 11-$\beta$-hydroxylation reactions in the adrenal cortex, markedly reduces the secretion of cortisol, corticosterone and aldosterone, while the secretion of 11-deoxycorticosteroids is relatively unimpaired. Accordingly, the administration of methyrapone results in a compensatory increase in the secretion of ACTH and in enhanced secretion of 11-deoxycortisol, a relatively inert steroid, and 11-deoxycorticosterone, a mineralocorticoid. Therefore, methyrapone blocks the production of glucocorticoids but does not typically cause a deficiency of mineralocorticoids.

Under the influence of local inflammatory stimuli, cells emerge into the perivascular tissue. The number of cells, chiefly neutrophils, present in a standard area in the tissue is greater in adrenalectomized than in control animals. Endogenous glucocorticoids, therefore, appear to control leukocyte migration to an inflamed area through an interference with leukocyte–endothelium interactions.[65] Adhesion glycoproteins of the integrin family ($\beta 2$ integrins), collectively termed CD11/CD18 complex,[134,135] are only expressed in haematopoietic precursor cells and in white blood cells. The complex is composed of $\alpha\beta$ glycoprotein heterodimers with distinct $\alpha$ chains (CD11a, CD11b, CD11c), and a common $\beta$ subunit (CD18). Administration of a monoclonal antibody anti-CD18 to adrenalectomized rats completely blocks the previously enhanced migration of leukocytes from the microcirculation to the perivascular inflamed tissue. The finding suggests that glucocorticoids may govern the expression or the activity of the CD11/CD18 complex on the surface of leukocytes, thereby limiting the migration of these cells to inflamed areas.[136]

*Glucocorticoids* vs *insulin:* A possible interrelationship may exist between endogenous glucocorticoids and insulin in controlling the development of acute inflammation.[127] Insulin appears to facilitate the action of vasoactive substances on vascular endothelial cells, thus contributing to the formation of interendothelial gaps and to increased vascular permeability to plasma proteins. The activation of the hypothalamus–pituitary–adrenal axis by factors formed in inflamed tissue results in increased levels of circulating glucocorticoids which, in turn, are relevant for the maintenance of the integrity of the microcirculation. The immediate opening of the endothelial intercellular junctions at the venular side of the microvasculature produced by inflammatory stimuli is a transient event, even in the continuous presence of permeability-increasing agents.[137] It is plausible, therefore, to suggest that increased concentrations of circulating glucocorticoids, as observed during the early stages of the inflammatory response, might antagonize the facilitatory effect of insulin upon endothelial cells of the reacting vessels, thereby limiting the development of the response.

*Lipocortins:* Lipocortin has been described as a glycoprotein whose synthesis or secretion is stimulated by glucocorticoids through receptor-mediated actions and which specifically inhibits phospholipase $A_2$ *in vitro* and *in vivo*.[138] It is now known that this substance belongs to a much larger class of proteins which share the property of binding membrane phospholipids in a calcium-dependent manner. Due to inhibition of the phospholipase $A_2$ activity, a controlling enzyme in the production of eicosanoids and PAF, lipocortins have been suggested to act as mediators of the anti-inflammatory effect of glucocorticoids. More recently, the relevance of the inhibitory action of lipocortins on phospholipase $A_2$ activity has been questioned.[139,140] Lipocortins are thought to act extracellularly and must, therefore, be secreted but there is no direct evidence that they are, in fact, secreted proteins and it is not known whether they act as inhibitors of intracellular phospholipase. Purified lipocortins block the stimulated release of arachidonic acid from membrane phospholipids, but not basal release. It is not clear whether any of the recombinant or otherwise structurally characterized lipocortins are actually induced by glucocorticoids particularly in a way that correlates with anti-inflammatory activity. However, the possibility has been raised that certain proteins can be secreted by pathways other than those involving the trans-Golgi network. Following the recent discovery of putative receptors on phagocytes for lipocortins (lipocortin-1), and the observation that these receptors influence the inflammatory actions of the cells by mechanisms independent of inhibition of phospholipase $A_2$ activity, a novel interpretation for the role of lipocortins on inflammation has been proposed. Activation of the hypothalamus–pituitary–adrenal axis by potential mediators, including interleukins and TNF, during the early stages of the inflammatory response would result in an enhanced secretion of glucocorticoids, as referred to above. This in turn, would lead to the production of lipocortins at discrete sites throughout the body. Saturation of lipocortin-binding sites in phagocytes would then reduce the migratory and proinflammatory activity of these cells with a consequent downregulation of the host response.[141] Apparently, even physiological levels of lipocortin 1 in the absence of inflammation are regulated by glucocorticoids.[142]

*The anti-inflammatory action of glucocorticoids: molecular mechanisms:* As for most biological effects of steroids, the anti-inflammatory activity of glucocorticoids involves receptor occupancy and induction of gene expression. The molecular mechanisms underlying this activity were established by two lines of evidence. One derives from experiments in which

**Table 2.** Evidence for the role of glucocorticoids as modulators of inflammatory responses

| | Reference no. |
|---|---|
| Occurrence of specific receptors in endothelial and inflammatory cells. | 96,97,114,143,145,146 |
| Increased concentrations of circulating glucocorticoids at the early stages of inflammation resulting in downregulation of inflammatory responses. | 126–128 |
| Enhanced microvascular responses to inflammatory mediators and increased migration of cells to inflamed areas in adrenalectomized animals. | 65,91,126 |
| Potent anti-inflammatory and immunosuppressive effects following exogenous administration: | |
| (a) Reduction of cytokine production or activity; | 105–108,120–123 |
| (b) Decreased microvascular responses to inflammatory mediators; | 91–95 |
| (c) Inhibition of leukocyte accumulation at inflamed sites; | 110,111 |
| (d) Blockade of phagocytic functions and impairment of microbicidal capacity of polymorphonuclear leukocytes; | 112,113 |
| (e) Inhibition of recruitment of mononuclear phagocytes to injured areas; | 112,113 |
| (f) Interference with leukocyte kinetics; | 112,114–116 |
| (g) Interference with the activity of the immune system. | 114,116,119,121–123 |

inhibition of RNA and protein synthesis were used to study the effects of dexamethasone. The existence of a time lag for manifestation of the anti-inflammatory effect of the steroid was interpreted to represent time required to bring about production of specific proteins through gene expression. The effect is suppressed by actinomycin D or cycloheximide, thereby indicating that RNA and protein synthesis are essential for the expression of the anti-inflammatory activity of the glucocorticoid.[143] The other line of evidence is based on the effects of RU38486, a synthetic glucocorticoid receptor antagonist. The substance exhibits a high affinity for the corresponding receptor and displays antiglucocorticoid activity *in vivo* and *in vitro*.[144] Treatment with RU38486 blocks, in a dose-dependent manner, the anti-inflammatory effect of dexamethasone under various experimental conditions, confirming that this action, like most steroid actions, occurs by receptor-mediated processes.[145,146]

*Conclusions:* Glucocorticoids are potent anti-inflammatory agents and have long been used as valuable medications in the management of a variety of diseases. Usually, analogues of cortisol, the prevalent glucocorticoid in humans, are employed in glucocorticoid therapy. In the last two decades evidence has been obtained that the self-limiting character of inflammatory responses is associated with increased plasma concentrations of the steroids. The increased secretion, induced via the hypothalamus–pituitary–adrenal axis by putative inflammatory mediators, represents the last event of a feedback mechanism that regulates the development of inflammation. Glucocorticoids are relevant for the maintenance of the functional integrity of the microcirculation, and exert profound effects on inflammatory cell functions. Furthermore, they apparently govern leukocyte–endothelial interactions in inflammation and the subsequent migration of haematogenous cells to perivascular inflamed tissues. Evidence supporting the involvement of glucocorticoids in the control of inflammation is summarized in Table 2.

### Oestrogens

Granuloma formation and croton oil-induced inflammation in experimental animals are lessened by oestrogen treatment.[147,148] Oestrogens suppress rat paw oedemas irrespective of the inducer irritant,[149] and exhibit antipermeability effects on skin vessels.[150] Oestradiol consistently diminishes the incidence of inflammatory joint disease evoked by adjuvant injected in rats. This protective effect is apparent over a wide dose range and in a variety of treatment schedules. Moreover, estimation of the severity of the arthritis indicates that oestradiol-treated animals have a much milder disease as compared with controls. Oestrone and oestriol are as effective in suppressing arthritis as the parent compound.[151] Oestrogens markedly reduce the cutaneous inflammatory response to tuberculin in rabbits sensitized by active turberculosis or by treatment with heat-killed tubercle bacilli. Further, although oestrogen-treated rabbits develop the same number of pulmonary lesions after inhalation of tubercle bacilli, the size of the lesions is significantly smaller in the treated group.[151] Oestrogens enhance anti-inflammatory actions of corticosteroids in the treatment of inflammatory skin disease in humans.[147]

The mechanism through which oestrogens influence acute inflammation is not clear. A protecting activity on microvessels which is independent of a systemic action is suggested, since the effect is observed by direct application of the hormones to the target tissue.[152,153] This has been thought to be accomplished through an influence on acid mucopolysaccharides of the perivascular ground substance or through the activation of the adenylate cyclase system that regulates leukocytic lysosomal enzyme release.[154–156] The mechanism by which oestradiol, oestrone and oestriol confer protection in adjuvant arthritis is also unknown. Since lymphocytes are involved in the pathogenesis of this disease, the well-known depleting effects of oestrogens on lymphoid tissues might account in part for the protective action of these hormones. However, the finding that the hormones diminish the incidence of arthritis when administered in the latent period either starting on the day of inoculation, or 5–10 days thereafter, associated with the fact that the immunological reaction leading to arthritis is quickly initiated, suggest a direct hormonal effect on the capacity of involved tissues to react to the inflammatory stimulus in this disorder.[151]

Oestrogens are reported to influence antibody formation, and to suppress cell mediated immunity. In addition, the hormones inhibit lymphocyte responses to nonspecific mitogens.

Effects of oestradiol and related steroids on leukocyte function and metabolism *in vitro* have been investigated. Human blood leukocytes, incubated with oestradiol for 1 h before the addition of live *Staphylococci,* ingest the same number of bacteria as do control leukocytes. The normal increase in oxygen consumption after phagocytosis, however, is reduced in the presence of oestradiol. Cells incubated with oestradiol have diminished granule lysis after phagocytosis.[157] Oestrogens, apparently influence the microbicidal actvity of the myeloperoxidase system in polymorphonuclear leukocytes.[158] Accordingly, changes in the hormonal environment involving oestrogens may modify leukocyte responses to inflammatory stimuli. Further information, however, is required for a clear interpretation of the way these hormones may act.

### Indirectly acting hormones

*Glucagon:* Any stimulus of sufficient intensity to constitute a stress and cause a nonspecific increase in sympathetic efferent activity is likely to produce a rise in plasma glucagon concentration.[159] Plasma glucagon levels are elevated in patients with bacterial infections, in burns, after endotoxin administration, or following injection of bacterial pyrogens.[159–161] Glucagon interferes with the development of inflammatory responses. Given daily by the subcutaneous route, after a local reaction to Freund's adjuvant has already developed in the rat, glucagon produces a decrease in the response, comparable to that attained with daily effective doses of indomethacin. Administered subcutaneously 30 min beforehand, it reduces oedema formation in the 4-h interval which follows the injection of chemical irritants into the rat's paw, and decreases the local exudation of Evans blue previously given intravenously. Apparently this is an indirect effect since it is not observed in adrenalectomized animals, but persists after the removal of the adrenal medulla.[162] It is improbable that metabolic changes may play a role in this circumstance, because increased insulin secretion can quickly compensate for and oppose the effects of glucagon.

The investigation of possible effects of glucagon on inflammatory cells has received limited attention despite the fact that hormones that influence carbohydrate metabolism are intimately involved in the host defence. During the early febrile phases of bacterial and viral illnesses, fasting plasma concentrations of glucose, insulin, glucagon, and even growth hormone tend to be increased. If carbohydrate tolerance is measured by means of an intravenous glucose load at the time, glucose disappearance rates show changes approaching those characteristic of maturity onset diabetes. Furthermore, concurrent hypercortisolism, documented in clinical studies by increased urinary excretion of free cortisol during fever, has been shown to influence glucose tolerance deleteriously not only through enhancement of gluconeogenesis, but also through augmentation of glucagon secretion.[163,164] This interplay of hormone actions is an integral part of the systemic response of the host against invading microorganisms and may serve a useful purpose in terms of adaptative metabolic changes, cell kinetic alterations, and energy supply during infection. The increased activity of pancreatic islets may reflect, therefore, the need for homeostatic adjustments in this condition.

Human mononuclear leukocytes specifically bind glucagon. The molecular basis for the actions of this hormone in target tissues is the activation of the adenylate cyclase system in plasma membranes following the interaction with specific macromolecular receptors. Elevation of intracellular concentrations of cAMP appears to mediate the various physiological actions of glucagon. Its effects on mononuclear leukocytes, however, are much less pronounced than in liver plasma membranes. To date, there is no indication of a possible involvement of glucagon in the metabolism of peripheral leukocytes. Therefore, the significance of its binding to mononuclear leukocytes remains uncertain.[165]

*Thyroid hormones:* Conflicting results have been obtained from investigations on the influence of thyroid gland on inflammation. This might reflect the multiplicity of effects of thyroid hormones on metabolic pathways, enzyme activities, and on the response of target tissues to other hormones. In addition, the age of the animals at which the hormonal disorder is produced and the duration of the prevailing alteration might be factors in the variation of the results observed. Nevertheless, there is evidence to show that altered inflammatory responses occur in hyperthyroid states.

In an attempt to identify the mechanisms through which thyroid gland activity can influence the development of inflammatory reactions, the capacity to respond to noxious stimuli was tested in rats when thyroid defects induced by hormone administration of thyroidectomy, were fully established. Whereas animals kept in a condition of sustained excess of circulating thyroid hormones present consistently depressed inflammatory responses, hypothyroid rats respond in a normal fashion.[166,167] Decreased reactions to intracutaneously injected histamine and serotonin; inhibited swelling response to carrageenan injected into the paws; and depressed primary and secondary reactions to Freund's adjuvant only occur in the group of hormone-treated animals. In addition, enlargement of the adrenal glands; reduced content of adrenal ascorbic acid; and decreased number of circulating eosinophils, which characterize a circumstance of adrenal cortical hyperactivity, are observed only in this group. A spontaneous reversal of the previously inhibited acute inflammatory response to carrageenan occurs 3–4 days after interruption of hormone administration to the animals and this is coincidental with the return to normal of the formerly enlarged adrenal glands. Similarly, specific inhibition of adrenal cortical steroid biosynthesis in hormone-treated animals with aminoglutethimide restores the previously depressed response to carrageenan without interference with the increased levels of serum thyroxine.[167] The findings suggest that the inhibitory effects of thyroid hormones on inflammatory responses are likely to be indirect.

There are considerable interactions between the activities of the thyroid gland, the adrenal cortex and pancreatic islet B-cells. Hypertrophy of the adrenals and reduced content of adrenal ascorbic acid are observed following thyroid hormone administration,[168–170] whereas thyroidectomy or injection of antithyroid drugs alone may result in atrophy of the adrenal gland.[171,172] The influence of thyroid hormones on the activity of the adrenal gland is either direct or indirect through changes in the release of ACTH.[173–175] Pancreatic B-cell dysfunction has been suggested by observations of decreased insulin secretion in isolated islets from animals made thyrotoxic, and by either decreased plasma insulin responses to an intravenous glucose challenge or lack of increased insulin levels in the face of hyperglycaemia.[176–178] Such interactions are a reasonable explanation for the effects of thyroid hormones on inflammation.

### Concluding remarks

Almost any stage of inflammatory and immunological responses are affected by hormone actions. Pro- and anti-inflammatory effects are recognized and this forms the basis for the suggestion that hormones exert an integrative function on inflammation. The effect of a hormone ultimately depends on its interaction with a receptor, and a major determinant of this step is the circulating level of the hormone. Specific hormone receptors are demonstrable in the reacting structures of an inflamed area. Endocrine disorders alter host defences against trauma and infection. Inflammation, therefore, is a hormone-controlled process. Consequently, management of inflammatory diseases should take into account the general condition of the patient in addition to the clinical evaluation of local signs and symptoms.

### References

1. Miles AA, Niven JSF. The enhancement of infection during schock produced by bacterial toxins and other agents. *Br J Exp Pathol* 1950; **31:** 73–80.
2. Miles AA. Nonspecific defense reactions in bacterial infections. *Ann NY Acad Sci* 1956; **66:** 356–361.
3. Miles AA, Miles EM, Burke JF. The value and duration of defense reactions of the skin to the primary lodgement of bacteria. *Br J Exp Pathol* 1957; **38:** 79–83.
4. Leak LV, Burk JF. Early events of tissue injury and the role of the lymphatic system in early inflammation. In: Zweifach BW, Grant L, McCluskey RT, eds. *The Inflammatory Process*, vol. 3, 2nd edition. New York: Academic Press, 1974; 163–265.
5. Wilhelm DL. Chemical mediators. In: Zweifach BW, Grant L, McCluskey RT, eds. *The Inflammatory Process*, vol. 2, 2nd edition. New York: Academic Press, 1973; 251–301.
6. Willoughby DA. Mediation of increased vascular permeability in inflammation. In: Zweifach BW, Grant L, McCluskey RT, eds. *The Inflammatory Process*, vol. 2, 2nd edition. New York: Academic Press, 1973; 303–331.
7. Bonta IL. Endogenous modulators of the inflammatory response. In: Vane JR, Ferreira SH, eds. *Inflammation* (*Handbook of Experimental Pharmacology 50/1*). Berlin: Springer-Verlag, 1978; 523–567.
8. Garcia-Leme J. Regulatory mechanisms in inflammation: new aspects of autopharmacology. *Gen Pharmacol* 1981; **12:** 15–24.
9. Garcia-Leme J. The endocrine and nervous system in inflammation: pharmacological considerations. In: Bonta IL, Bray MA, Parnham MJ, eds. *The Pharmacology of Inflammation* (*Handbook of Inflammation,* vol. 5). Amsterdam: Elsevier, 1985; 195–234.
10. Garcia-Leme J. *Hormones and Inflammation.* Boca Raton: CRC Press, 1989.
11. Freychet P, Kahn CR, Jarrett DB, Roth J. Insulin receptors in the liver: specific binding of $^{125}$I-insulin to the plasma membrane and its relations to insulin bioactivity. *Proc Natl Acad Sci USA* 1971; **68:** 1833–1837.
12. Cuatrecasas P. Insulin receptor interactions in adipose tissue cells: direct measurement and properties. *Proc Natl Acad Sci USA* 1971; **68:** 1264–1268.
13. Kono T, Barham FW. The relationship between the insulin-binding capacity of fat cells and the cellular response to insulin. *J Biol Chem* 1971; **246:** 6210–6216.
14. Haring HU. The insulin receptor: signalling mechanism and contribution to the pathogenesis of insulin resistance. *Diabetologia* 1991; **34:** 848–861.
15. Olefsky JM. Effect of dexamethasone on insulin binding, glucose transport and glucose oxidation in isolated rat adipocytes. *J Clin Invest* 1975; **56:** 1499–1508.
16. Olefsky JM. The insulin receptor: its role in insulin resistance of obesity and diabetes. *Diabetes* 1976; **25:** 1154–1162.
17. Olefsky JM. Decreased insulin binding to adipocytes and circulating monocytes from obese subjects. *J Clin Invest* 1976; **57:** 1165–1172.
18. Pessin JE, Gitomer W, Oka Y, Oppenheimer CL, Czech MP. β-Adrenergic regulation of insulin and epidermal growth factor receptors in rat adipocytes. *J Biol Chem* 1983; **258:** 7386–7394.
19. Bar RS, Hoack JC, Peacock ML. Insulin receptors in human endothelial cells: identification and characterization. *J Clin Endocrinol Metab* 1978; **46:** 699–702.
20. Bar RS, Peacock ML, Spanheimer RG, Veenstra R, Hoack JC. Differential binding of insulin to human arterial and venous endothelial cells in primary culture. *Diabetes* 1980; **29:** 991–995.

21. Haskell JF, Meezan E, Pillon DJ. Identification of the insulin receptor of cerebral microvessels. *Am J Physiol* 1985; **248:** E115–125.
22. Corkey RF, Corkey BE, Gimbrone Jr MA. Hexose transport in normal and SV40-transformed human endothelial cells in culture. *J Cell Physiol* 1981; **106:** 425–434.
23. Meezan E, Pillion DJ. Direct demonstration that cerebral and retinal micro-vessels respond to insulin. Stimulation of glucose oxidation and phosphodiesterase activity. *Fed Proc (Abs)* 1981; **40:** 366.
24. Pillion DJ, Haskell JF, Meezan E. Cerebral cortical microvessels: an insulin-sensitive tissue. *Biochem Biophys Res Commun* 1982; **104:** 686–692.
25. Pfaffman MA, Ball CR, Darby A, Hilman R. Insulin reversal of diabetes-induced inhibition of vascular contractility in the rat. *Am J Physiol* 1982; **242:** H490–495.
26. McLeod KM. The effect of insulin treatment on changes in vascular reactivity in chronic experimental diabetes. *Diabetes* 1985; **34:** 1160–1167.
27. Fortes ZB, Scivoletto R, Garcia-Leme J. Functional changes in the micro-circulation of alloxan-induced diabetic rats. *Gen Pharmacol* 1989; **20:** 615–620.
28. Longhurst PA, Head RJ. Responses of the isolated perfused mesenteric vasculature from diabetic tissues: the significance of appropriate control tissues. *J Pharmacol Exp Ther* 1985; **235:** 45–49.
29. Fortes ZB, Garcia-Leme J, Scivoletto R. Influence of diabetes on the reactivity of mesenteric microvessels to histamine, bradykinin and acetylcholine. *Br J Pharmacol* 1983; **78:** 39–48.
30. Fortes ZB, Garcia-Leme J, Scivoletto R. Vascular reactivity in diabetes mellitus: role of the endothelial cell. *Br J Pharmacol* 1983; **79:** 771–781.
31. Vanhoutte PM. Heterogeneity in vascular smooth muscle. In: Kaley G, Altura BM, eds. *Microcirculation.* vol. 2. Baltimore: University Park Press, 1978; 181–309.
32. Fortes ZB, Garcia-Leme J, Scivoletto R. Vascular reactivity in diabetes mellitus: possible role of insulin on the endothelial cell. *Br J Pharmacol* 1984; **83:** 635–643.
33. Edlung T, Lofgren B, Vall L. Toxicity of dextran in rats. *Nature (Lond)* 1952; **170:** 125.
34. Goth A, Nash WL, Nagler M, Holman J. Inhibition of histamine release in experimental diabetes. *Am J Physiol* 1957; **191:** 25–28.
35. Adamkiewicz VW, Langlois Y. Sensitization by insulin to the dextran anaphylactoid reaction. *Can J Biochem Physiol* 1957; **35:** 251–256.
36. Llorach MAS, Bohm GM, Garcia-Leme J. Decreased vascular reaction to permeability factors in experimental diabetes. *Br J Exp Pathol* 1976; **57:** 747–754.
37. Majno G, Palade GE. Studies on inflammation. I. The effect of histamine and serotonin on vascular permeability. An electron microscopic study. *J Biophys Biochem Cytol* 1961; **11:** 571–605.
38. Majno G, Gilmore V, Leventhal M. On the mechanism of vascular leakage caused by histamine-type mediators. *Circ Res* 1967; **21:** 833–847.
39. Majno G, Shea SM, Leventhal M. Endothelial contraction induced by histamine-type mediators. An electron microscopic study. *J Cell Biol* 1969; **42:** 647–672.
40. Joris I, Majno G, Ryan GB. Endothelial interactions *in vivo:* a study of the rat mesentery. *Virchows Arch Cell Pathol* 1972; **12:** 73–83.
41. Morel NM, Petruzzo PP, Hechtman HB, Shepro D. Inflammatory agonists that increase microvascular permeability *in vivo* stimulate cultured pulmonary microvessel endothelial cell contraction. *Inflammation* 1990; **14:** 571–583.
42. Garcia-Leme J, Hamamura L, Migliorini RH, Leite MP. Influence of diabetes mellitus upon the inflammatory response of the rat. A pharmacological analysis. *Eur J Pharmacol* 1973; **23:** 74–81.
43. Garcia-Leme J, Bohm GM, Migliorini RH, Souza MZA. Possible participation of insulin in the control of vascular permeability. *Eur J Pharmacol* 1974; **29:** 298–306.
44. Gamse R, Jancsó G. Reduced neurogenic inflammation in streptozotocin-diabetic rats due to microvascular changes but not to substance P depletion. *Eur J Pharmacol* 1985; **118:** 175–180.
45. Bacala R, Cerami A. Advanced glycosylation: chemistry, biology, and implications for diabetes and ageing. *Adv Pharmacol* 1992; **23:** 1–34.
46. Gavin JR, Roth J, Jen P, Freychet P. Insulin receptors in human circulating cells and fibroblasts. *Proc Natl Acad Sci USA* 1972; **69:** 747–751.
47. Fussganger RD, Kahn CR, Roth J, De Meyts P. Binding and degradation of insulin by human peripheral granulocytes. Demonstration of specific receptors with high affinity. *J Biol Chem* 1976; **251:** 2761–2769.
48. Krug U, Krug F, Cuatrecasas P. Emergence of insulin receptors on human lymphocytes during *in vitro* transformation. *Proc Natl Acad Sci USA* 1972; **69:** 2604–2608.
49. Olefsky J, Reaven GM. The human lymphocyte: a model for the study of insulin-receptor interaction. *J Clin Endocrinol Metab* 1974; **38:** 554–560.
50. Gavin JR, Buell DN, Roth J. Water-soluble insulin receptors from human lymphocytes. *Science* 1972; **178:** 168–169.
51. Grunberger G, Robert A, Carpentier JL, *et al.* Human circulating monocytes internalize $^{125}$I-insulin in a similar fashion to rat hepatocytes: relevance to receptor regulation in target and nontarget tissues. *J Lab Clin Med* 1985; **106:** 211–217.
52. Livingston JN, Saran BR, Rose CD, Anderson CL. Rapid effects of insulin on the cycling of the insulin receptor in a human monocyte cell line (U-937). *Diabetes* 1985; **34:** 403–408.
53. Bar RS, Kahn CR, Koren HS. Insulin inhibition of antibody-dependent cytotoxicity and insulin receptors in macrophages. *Nature (Lond)* 1977; **265:** 632–635.
54. Hajek AS, Joist JH, Baker RK, Jarett L, Daughaday WH. Demonstration and partial characterization of insulin receptors in human platelets. *J Clin Invest* 1979; **63:** 1060–1065.
55. Esmann V. The diabetic leukocyte. *Enzyme* 1972; **13:** 32–55.
56. Savin JA. Bacterial infections in diabetes mellitus. *Br J Dermatol* 1974; **91:** 481–484.
57. Larkin JG, Frier BM, Ireland JI. Diabetes mellitus and infection. *Postgrad Med J* 1985; **61:** 233–237.
58. Kontras SB, Bodenbender MT. Studies of the inflammatory cycle in juvenile diabetes. *Am J Dis Child* 1968; **116:** 130–134.
59. Perrillie PE, Nolan JP, Finch SC. Studies of the resistance to infection in diabetes mellitus: local exudative cellular response. *J Lab Clin Med* 1962; **59:** 1008–1015.
60. Mowat AG, Baum J. Chemotaxis of polymorphonuclear leukocytes from patients with diabetes mellitus. *N Engl J Med* 1971; **284:** 621–624.
61. Miller ME, Baker L. Leukocyte functions in juvenile diabetes mellitus. Humoral and cellular aspects. *J Pediatr* 1972; **81:** 979–982.
62. Hill HR, Sauls HS, Dettloff JL, Quie PG. Impaired leukocyte responsiveness in patients with juvenile diabetes mellitus. *Clin Immunol Immunopathol* 1974; **2:** 395–403.
63. Mohandes AE, Touraine JL, Osman M, Salle B. Neutrophil chemotaxis in infants of diabetic mothers and in preterms at birth. *J Clin Lab Immunol* 1982; **8:** 117–120.
64. Pereira MAA, Sannomiya P, Garcia-Leme J. Inhibition of leukocyte chemotaxis by factor in alloxan-induced diabetic rat plasma. *Diabetes* 1987; **36:** 1307–1314.
65. Garcia-Leme J, Fortes ZB, Sannomiya P, Farsky SP. Insulin, glucocorticoids and the control of inflammatory responses. *Agents & Actions* 1992; **36:** 99–118.
66. Sannomiya P, Pereira MAA, Garcia-Leme J. Inhibition of leukocyte chemotaxis by serum factor in diabetes mellitus: selective depression of cell responses mediated by complement derived chemoattractants. *Agents & Actions* 1990; **30:** 369–376.
67. Firrel JC, Lipowsky HH. Leukocyte margination and deformation in mesenteric venules of rat. *Am J Physiol* 1989; **256:** H1667–1674.
68. Fortes ZB, Farsky SP, Oliveira MA, Garcia-Leme J. Direct vital microscopic study of defective leukocyte-endothelial interaction in diabetes mellitus. *Diabetes* 1991; **40:** 1267–1273.
69. Albelda SM, Buck CA. Integrins and other cell adhesion molecules. *FASEB J* 1990; **4:** 2868–2880.
70. Wertman KF, Henney MR. The effects of alloxan diabetes on phagocytosis and susceptibility to infection. *J Immunol* 1962; **89:** 314–317.
71. Drachman RH, Root RK, Wood Jr WB. Studies on the effect of experimental non-ketotic diabetes mellitus on antibacterial defense. I. Demonstration of a defect in phagocytosis. *J Exp Med* 1966; **124:** 227–240.
72. Tan JS, Anderson JL, Watanakunakorn C, Phair JP. Neutrophil dysfunctions in diabetes mellitus. *J Lab Clin Med* 1975; **85:** 26–33.
73. Bagdade JD. Phagocytic and microbicidal function in diabetes mellitus. *Acta Endocrinol.* 1976; **83** (suppl 205): 27–34.
74. Pozzilli P, Zuccarini O, Iavicolli M, *et al.* Monoclonal antibodies defined abnormalities of T-lymphocytes in Type I (insulin-dependent) diabetes. *Diabetes* 1983; **32:** 91–94.
75. Buschard K, Madsbad S, Rygaard J. Depressed suppressor cell activity in patients with newly diagnosed insulin-dependent diabetes mellitus. *Clin Exp Immunol* 1980; **41:** 25–32.
76. Fairchild RS, Kyner JL, Abdou NI. Specific immunoregulation abnormality in insulin-dependent diabetes mellitus. *J Lab Clin Med* 1982; **99:** 175–186.
77. Lederman MM, Ellner JJ, Rodman HM. Defective suppressor cell generation in juvenile onset diabetes. *J Immunol* 1981; **127:** 2051–2055.
78. Gupta S, Fikrig SM, Khanna S, Orti E. Deficiency of suppressor T-cells in insulin-dependent diabetes mellitus: an analysis with monoclonal antibodies. *Immunol Lett* 1982; **4:** 289–294.
79. MacCuish AC, Jordan J, Campbell CJ, Duncan LJP, Irvine WJ. Cell-mediated immunity in diabetes mellitus: lymphocyte transformation by insulin fragments in insulin-treated and newly diagnosed diabetics. *Diabetes* 1975; **24:** 36–43.
80. Zier KS, Leo MR, Spielman RS, Baker L. Decreased synthesis of interleukin-2 (IL-2) in insulin-dependent diabetes mellitus. *Diabetes* 1984; **33:** 552–555.
81. Rodman HM. Deficient interleukin-2 synthesis in type I diabetes. *Diabetes* 1984; **33:** 11A.
82. Dolkart RE, Halpern B, Perlman J. Comparison of antibody responses in normal and alloxan diabetic mice. *Diabetes* 1971; **20:** 162–167.
83. Vianna ESO, Garcia-Leme J. Unpublished results.
84. Munck A, Mendel DB, Smith LI, Orti E. Glucocorticoid receptors and actions. *Am Rev Respir Dis* 1990; **141:** S2–10.
85. Gustafsson JA, Carlstedt-Duke J, Poellinger L, *et al.* Biochemistry, molecular biology, and physiology of the glucocorticoid receptor. *Endocrinol Rev* 1987; **8:** 185–234.
86. Sherman MR, Stevens J. Structure of mammalian steroid receptors: evolving concepts and methodological developments. *Annu Rev Physiol* 1984; **46:** 83–105.
87. Miesfeld RL. Molecular genetics of corticosteroid action. *Am Rev Respir Dis* 1990; **141:** S11–17.

88. Yamamoto KR. Steroid receptor regulated transcription of specific genes and genes networks. *Annu Rev Genet* 1985; **19**: 209–252.
89. Litwach G. The glucocorticoid receptor at the protein level. *Cancer Res* 1988; **48**: 2636–2640.
90. Baxter JD. Minimizing the side effects of glucocorticoid therapy. *Adv Intern Med* 1990; **35**: 173–193.
91. Garcia-Leme J, Wilhelm DL. The effects of adrenalectomy and corticosterone on the vascular permeability responses in the skin of the rat. *Br J Exp Pathol* 1975; **56**: 402–407.
92. Inagaki N, Miura T, Nagai H, Ono Y, Koda A. Inhibition of vascular permeability increase in mice. An additional anti-allergic mechanism of glucocorticoids. *Int Arch Allergy Appl Immunol* 1988; **87**: 254–259.
93. Bjork J, Goldschmidt T, Smedegard G, Arfors KE. Methylprednisolone acts at the endothelial cell level reducing inflammatory responses. *Acta Physiol Scand* 1985; **123**: 221–223.
94. Svensjo E, Roempke K. Time-dependent inhibition of bradykinin- and histamine-induced microvascular permeability increase by local glucocorticoid treatment. *Prog Respir Res* 1985; **19**: 173–180.
95. Erlansson M, Svensjo E, Bergqvist D. Leukotriene $B_4$-induced permeability increase in postcapillary venules and its inhibition by three different anti-inflammatory drugs. *Inflammation* 1989; **13**: 693–705.
96. Crutchley DJ, Ryan US, Ryan JW. Glucocorticoid modulation of prostacyclin production in cultured bovine pulmonary endothelial cells. *J Pharmacol Exp Ther* 1985; **233**: 650–655.
97. Maca RD, Fry GL, Hoack JC. The effects of glucocorticoids on cultured human endothelial cells. *Br J Haematol* 1978; **38**: 501–509.
98. Tonnesen MG, Smedley L, Henson PM. Neutrophil–endothelial cell interactions. Modulation of neutrophil adhesiveness induced by complement fragments C5a and C5a des Arg and formyl-methionyl-leucyl-phenylalanine *in vitro*. *J Clin Invest* 1984; **74**: 1581–1592.
99. Moser R, Schleiffenbaum B, Groscurth P, Fehr J. Interleukin 1 and tumor necrosis factor stimulate human vascular endothelial cells to promote transendothelial neutrophil passage. *J Clin Invest* 1989; **83**: 444–455.
100. Gamble JR, Harlan JM, Klebanoff SJ, Vadas MA. Stimulation of the adherence of neutrophil to umbilical vein endothelium by human recombinant tumor necrosis factor. *Proc Natl Acad Sci USA* 1985; **82**: 8667–8671.
101. Lo SK, Detmers PA, Levin SM, Wright SD. Transient adhesion of neutrophils to endothelium. *J Exp Med* 1989; **169**: 1779–1793.
102. Lopez AJ, Williamson DJ, Gamble JR, *et al.* Recombinant human granulocyte-macrophage-colony-stimulating factor (rH GM-CSF) stimulates *in vitro* mature human neutrophil and eosinophil function, surface receptor expression and survival. *J Clin Invest* 1986; **78**: 1220–1228.
103. Bevilacqua MP, Pober JS, Wheeler ME, Cotran RS, Gimbrone Jr MA. Interleukin 1 acts on cultured human vascular endothelium to increase the adhesion of polymorphonuclear leukocytes, monocytes and related leukocyte cell lines. *J Clin Invest* 1985; **76**: 2003–2011.
104. Dinarello CA, Mier JW. Lymphokines. *N Engl J Med* 1987; **317**: 940–945.
105. Beutler B, Cerami A. Cachetin: more than a tumor necrosis factor. *N Engl J Med* 1987; **316**: 379–385.
106. Staruch MJ, Wood DD. Reduction of serum interleukin-1-like activity after treatment with dexamethasone. *J Leukocyte Biol* 1985; **37**: 193–207.
107. Snyder DS, Unanue ER. Corticosteroids inhibit murine macrophage Ia expression and interleukin 1 production. *J Immunol* 1982; **129**: 1803–1805.
108. Bochner BS, Rutledge BK, Schleimer RP. Interleukin 1 production by human lung tissue. II. Inhibition by anti-inflammatory steroids. *J Immunol* 1987; **139**: 2303–2307.
109. Schleimer RP, Freeland HS, Peters SP, Brown KE, Derse CP. An assessment of the effects of glucocorticoids on degranulation, chemotaxis, binding to vascular endothelium and formation of leukotriene $B_4$ by purified human neutrophils. *J Pharmacol Exp Ther* 1989; **250**: 598–605.
110. Ishikawa W, Mori Y, Tsurufuji S. The characteristic feature of glucocorticoids after local application with reference to leukocyte migration and protein exudation. *Eur J Pharmacol* 1969; **7**: 201–205.
111. Perper RJ, Sanda M, Chinea G, Ornsky AL. Leukocyte chemotaxis *in vivo*. II. Analysis of the selective inhibition of neutrophil on mononuclear cell accumulation. *J Lab Clin Med* 1974; **84**: 394–406.
112. Mishler JM. The effects of corticosteroids on mobilization and function of neutrophils. *Exp Hematol* 1977; **5** (suppl): 15–32.
113. Baxter JD. Glucocorticoid hormone action. *Pharmacol Ther B* 1976; **2**: 605–659.
114. Claman HN. Corticosteroids and lymphoid cells. *N Engl J Med* 1972; **287**: 388–397.
115. Fauci AS, Dale DC. The effect of *in vivo* hydrocortisone on subpopulations of human lymphocytes. *J Clin Invest* 1974; **53**: 240–246.
116. Parrillo JE, Fauci AS. Mechanisms of glucocorticoid action on immune processes. *Annu Rev Pharmacol Toxicol* 1979; **19**: 179–201.
117. Butler WT. Corticosteroids and immunoglobulin synthesis. *Transplant Proc* 1975; **7**: 49–53.
118. Claman HN. How corticosteroids work. *J Allergy Clin Immunol* 1975; **55**: 145–151.
119. Balow JE, Rosenthal AS. Glucocorticoid suppression of macrophage migration inhibitory factor. *J Exp Med* 1973; **137**: 1031–1041.
120. Lew W, Oppenhein JJ, Matsushima K. Analysis of the suppression of IL-1$\alpha$ and IL-1$\beta$ production in human peripheral blood mononuclear adherent cells by a glucocorticoid hormone. *J Immunol* 1988; **140**: 1895–1902.
121. Gillis S, Crabtree GR, Smith KA. Glucocorticoid-induced inhibition of T-cell growth factor production. I. The effect on mitogen-induced lymphocyte proliferation. *J Immunol* 1979; **123**: 1624–1631.
122. Gillis S, Crabtree GR, Smith KA. Glucocorticoid-induced inhibition of T-cell growth factor. II. The effect on the *in vitro* generation of cytolytic T-cells. *J Immunol* 1979; **123**: 1632–1638.
123. Reed JC, Abidi AH, Alpers JD, Hoover RG, Robb RJ, Nowell PC. Effect of cylcosporin A and dexamethasone on interleukin-2 receptor gene expression. *J Immunol* 1986; **137**: 150–154.
124. Melby J, Egdahl R, Spink W. Secretion and metabolism of cortisol after injection of endotoxin. *J Lab Clin Med* 1960; **56**: 50–62.
125. Moberg GP. Site of action of endotoxins on hypothalamic–pituitary–adrenal axis. *Am J Physiol* 1971; **220**: 397–400.
126. Garcia-Leme J, Schapoval EES. Stimulation of the hypothalamic–pituitary–adrenal axis by compounds formed in inflamed tissue. *Br J Pharmacol* 1975; **53**: 75–83.
127. Moraes FR, Garcia-Leme J. Endogenous corticosteroids and insulin in acute inflammation. *Microvasc Res* 1982; **23**: 281–293.
128. Stenberg VI, Bouley MG, Katz BM, Lee KJ, Parmar SS. Negative endocrine control system for inflammation in rats. *Agents & Actions* 1990; **29**: 189–195.
129. Sternberg EM, Hill JM, Chrousos GP, *et al.* Inflammatory mediator-induced hypothalamic–pituitary–adrenal axis activation is defective in streptococcal cell wall arthritis-susceptible lewis rats. *Proc Natl Acad Sci USA* 1989; **86**: 2374–2378.
130. Besedovsky H, Del Rey A, Sorkin E, Dinarello CA. Immunoregulatory feedback between interleukin 1 and glucocorticoid hormones. *Science* 1986; **233**: 652–654.
131. Sapolsky R, Rivier C, Yamamoto G, Plotstky P, Vale W. Interleukin 1 stimulates the secretion of hypothalamic corticotropin releasing factor. *Science* 1987; **238**: 522–524.
132. Bateman A, Singh A, Krai T, Solomon S. The immune-hypothalamic–pituitary–adrenal axis. *Endocr Rev* 1989; **10**: 92–112.
133. Atherton A, Born GVR. Quantitative investigations of the adhesiveness of circulating polymorphonuclear leukocytes to blood vessel walls. *J Physiol* 1972; **222**: 447–474.
134. Arnaout MA. Leukocyte adhesion molecules deficiency: its structural basis, pathophysiology and implications for modulating the inflammatory response. *Immunol Revs* 1990; **114**: 145–179.
135. Anderson DC, Springer TA. Leukocyte adhesion deficiency: an inherited defect in the Mac-1, LFA-1 and p150,95 glycoproteins. *Annu Rev Med* 1987; **38**: 175–194.
136. Farsky SP, Sannomiya P, Garcia-Leme J. Unpublished results.
137. Casley-Smith JR, Windey J. Quantitative morphological correlations in capillary permeability following histamine and moderate burning in the mouse diaphragm and the effects of benzopyrones. *Microvasc Res* 1976; **11**: 279–305.
138. Flower RJ. Background and discovery of lipocortins. *Agents & Actions* 1985; **17**: 255–262.
139. Davidson FF, Dennis EA. Biological relevance of lipocortins and related proteins as inhibitors of phospholipase $A_2$. *Biochem Pharmacol* 1989; **38**: 3645–3651.
140. Whitehouse BJ. Lipocortins, mediators of the anti-inflammatory action of corticosteroids? *J Endocrinol* 1989; **123**: 363–366.
141. Goulding NJ, Guyre PM. Regulation of inflammation by lipocortin 1. *Immunol Today* 1992; **13**: 295–297.
142. Vishwanath BS, Frey FJ, Bradbury M, Dallman MF, Frey BM. Adrenalectomy decreases lipocortin-1 messenger ribonucleic acid and tissue protein content in rats. *Endocrinology* 1992; **130**: 585–591.
143. Tsurufuji S, Sugio K, Takemasa F. The role of glucocorticoid receptor and gene expression in the anti-inflammatory action of dexamethasone. *Nature (Lond)* 1979; **280**: 408–410.
144. Bertagna X, Bertagna C, Luton JP, Husson JM, Girard F. The new steroid analog RU 38486 inhibits glucocorticoid action in man. *J Clin Endocrinol Metab* 1984; **59**: 25–28.
145. Laue L, Kawai S, Brandon DD, *et al.* Receptor-mediated effects of glucocorticoids on inflammation: enhancement of the inflammatory response with a glucocorticoid antagonist. *J Steroid Biochem* 1988; **29**: 591–598.
146. Peers SH, Moon D, Flower RJ. Reversal of the anti-inflammatory effects of dexamethasone by the glucocorticoid antagonist RU 38486. *Biochem Pharmacol* 1988; **37**: 556–557.
147. Spangler AS, Antoniades HN, Sotman SL. Enhancement of the anti-inflammatory action of hydrocortisone by estrogen. *J Clin Endocrinol Metab* 1969; **29**: 650–655.
148. Glenn EM, Miller WL, Schlegel CA. Metabolic effects of adrenocortical steroids *in vivo* and *in vitro*: relationship to anti-inflammatory effects. *Rec Progr Horm Res* 1963; **19**: 107–199.
149. Bonta IL, De Vos CJ. The effect of estriol-16,17-dihemisuccinate on vascular permeability as evaluated in the rat paw oedema test. *Acta Endocrinol* 1965; **49**: 403–411.
150. Ishioka T, Honda Y, Sagara A, Shimamoto T. The effect of oestrogens on blueing lesions by bradykinin and histamine. *Acta Endocrinol* 1969; **60**: 177–183.
151. Mueller MN, Kappas A. Estrogen pharmacology. II. Suppression of experimental immune polyarthritis. *Proc Soc Exp Biol Med* 1964; **117**: 845–847.

152. Bonta IL, De Vos CJ, Delver A. Inhibitory effects of oestriol-16-17-disodium succinate on local haemorrhages induced by snake venon in canine heart-lung preparations. *Acta Endocrinol* 1965; **48**: 137–146.
153. Bonta IL, Vargaftig BB, De Vos CJ, Grijsen H. Haemorrhagic mechanisms of some snake venoms in relation to protection by estriol succinate of blood vessel damage. *Life Sci* 1969; **8**: 881–888.
154. Vincent JE, Bonta IL, De Vries-Kragt K, Bhargava N. L'influence des oestrogènes sur la perméabilité de la paroi vasculaire. *Steroidologia* 1970; **1**: 367–377.
155. Hempel KH, Fernandez LA, Persellin RH. Effect of pregnancy sera on isolated lysosomes. *Nature (Lond)* 1970; **225**: 955–956.
156. Persellin RH, Vance SE, Perry A. Effect of pregnancy serum on experimental inflammation. *Br J Exp Pathol* 1974; **55**: 26–32.
157. Bodel P, Dillard GM, Kaplan SS, Malawista SE. Anti-inflammatory effects of oestradiol on human blood leukocytes. *J Lab Clin Med* 1972; **80**: 373–384.
158. Klebanoff SJ. Effect of estrogens on the myeloperoxidase-mediated antimicrobial system. *Infect Immun* 1979; **25**: 153–156.
159. Bloom SR. Glucagon, a stress hormone. *Postgrad Med J* 1973; **49**: 607–611.
160. Santeusanio F, Rocha DM, Faloona GR, Muller W, Unger RH. The role of glucagon in the worsening of the diabetic state induced by infection. *Diabetes* 1972; **21** (suppl 1): 324–329.
161. Lindsey CA, Willmore DW, Moylan JA, Faloona GR, Unger RH. Glucagon and the insulin: glucagon (I : G) ratio in burns and trauma. *Clin Res* 1972; **20**: 802–805.
162. Garcia-Leme J, Morato M, Souza MZA. Anti-inflammatory action of glucagon in rats. *Br J Pharmacol* 1975; **55**: 65–68.
163. Beisel WR. Metabolic response to infection. *Annu Rev Med* 1975; **26**: 9–20.
164. Rayfield EJ, Curnow RT, George DT, Beisel WR. Impaired carbohydrate metabolism during a mild viral illness. *N Engl J Med* 1973; **289**: 618–621.
165. Blecher M, Goldstein S. Hormone receptors. VI. On the nature of the binding of glucagon and insulin to human circulating mononuclear leukocytes. *Mol Cell Endocrinol* 1977; **8**: 301–315.
166. Sena L, Torrielli MW, Franzone J, Curzio M, Cirillo R. The influence of experimental hypo- and hyperthyroid states on acute and chronic inflammatory reactions: modified response to non-steroidal anti-inflammatory agents. *J Pathol Bacteriol* 1981; **135**: 9–17.
167. Cury Y, Garcia-Leme J. The inflammatory response of hyperthyroid and hypothyroid rats. Role of adrenocortical steroids. *Agents & Actions* 1984; **15**: 377–385.
168. Wallach DP, Reineke EP. The effect of varying levels of thyroidal stimulation on the ascorbic acid content of the adrenal cortex. *Endocrinology* 1949; **45**: 75–81.
169. Timiras PS, Woodbury DM. Adrenocortical function after administration of thyroxine and triiodothyronine in rats. *J Pharmacol Exp Ther* 1955; **115**: 144–153.
170. Steinetz BG, Beach VL. Some influences of thyroid on the pituitary–adrenal axis. *Endocrinology* 1963; **72**: 45–78.
171. Zarrow MX, Horger LM, McCarthy JL. Atrophy of adrenal gland following thiouracil and vit. B12. *Proc Soc Exp Biol Med* 1957; **94**: 348–349.
172. Gaunt R, Gisoldi E, Steinetz BG, Chart JJ. Effect of thyroidectomy and aminoglutethimide on adrenal function in rats. *Endocrinology* 1970; **87**: 1088–1090.
173. Earthy H, Leblond CP. Identification of the effects of thyroxin mediated by the hypophysis. *Endocrinology* 1959; **54**: 249–271.
174. Moore NA, Boler RK. A comparative morphometric analysis of the effects of thyroxine and ACTH on the zona fasciculata of rat adrenal cortex. *Am J Anat* 1976; **145**: 517–523.
175. Mengoli G, Montesi G, Lechi A, Rosa A, Bellotti G, Scuro LA. Influence of thyroid hormone on cortisol biosynthesis. A gas chromatographic analysis. *J Steroid Biochem* 1980; **13**: 445–447.
176. Malaisse W, Malaisse-Lagae F, McCraw E. Effects of thyroid function upon insulin secretion. *Diabetes* 1967; **16**: 643–646.
177. Andreani D, Menzingee G, Fallucca F, Aliberti G, Tamburrano G, Cassano C. Insulin levels in thyrotoxicosis and primary myxoedema: response to intravenous glucose and glucagon. *Diabetologia* 1970; **6**: 1–7.
178. Asano T, Okumura M. Insulin delivery rate in response to glucose and arginine infusion in hyperthyroidism. *Diabetologia* 1982; **23**: 108–113.

**Received 29 March 1993; accepted 1 April 1993**

## References

[PDF_01817] Albelda S. M., Buck C. A. (1990). Integrins and other cell adhesion molecules.. FASEB J.

[PDF_01990] Anderson D. C., Springer T. A. (1987). Leukocyte adhesion deficiency: an inherited defect in the Mac-1, LFA-1, and p150,95 glycoproteins.. Annu Rev Med.

[PDF_02101] Andreani D., Menzinger G., Fallucca F., Aliberti G., Tamburrano G., Cassano C. (1970). Insulin levels in thyrotoxicosis and primary myxoedema: response to intravenous glucose and glucagon.. Diabetologia.

[PDF_01987] Arnaout M. A. (1990). Leukocyte adhesion molecules deficiency: its structural basis, pathophysiology and implications for modulating the inflammatory response.. Immunol Rev.

[PDF_02104] Asano T., Okumura M. (1982). Insulin delivery rate in response to glucose and arginine infusion in hyperthyroidism.. Diabetologia.

[PDF_01984] Atherton A., Born G. V. (1972). Quantitative investigations of the adhesiveness of circulating polymorphonuclear leucocytes to blood vessel walls.. J Physiol.

[PDF_02039] BONTA I. L., DE VOS C. J., DELVER A. (1965). INHIBITORY EFFECTS OF ESTRIOL-16,17-DISODIUM SUCCINATE ON LOCAL HAEMORRHAGES INDUCED BY SNAKE VENOM IN CANINE HEART-LUNG PREPARATIONS.. Acta Endocrinol (Copenh).

[PDF_02028] BONTA I. L., DE VOS C. J. (1965). THE EFFECT OF ESTRIOL-16,17-DIHEMISUCCINATE ON VASCULAR PERMEABILITY AS EVALUATED IN THE RAT PAW OEDEMA TEST.. Acta Endocrinol (Copenh).

[PDF_01826] Bagdade J. D. (1976). Phagocytic and microbicidal function in diabetes mellitus.. Acta Endocrinol Suppl (Copenh).

[PDF_01946] Balow J. E., Rosenthal A. S. (1973). Glucocorticoid suppression of macrophage migration inhibitory factor.. J Exp Med.

[PDF_01683] Bar R. S., Hoak J. C., Peacock M. L. (1978). Insulin receptors in human endothelial cells: identification and characterization.. J Clin Endocrinol Metab.

[PDF_01776] Bar R. S., Kahn C. R., Koren H. S. (1977). Insulin inhibition of antibody-dependent cytoxicity and insulin receptors in macrophages.. Nature.

[PDF_01686] Bar R. S., Peacock M. L., Spanheimer R. G., Veenstra R., Hoak J. C. (1980). Differential binding of insulin to human arterial and venous endothelial cells in primary culture.. Diabetes.

[PDF_01982] Bateman A., Singh A., Kral T., Solomon S. (1989). The immune-hypothalamic-pituitary-adrenal axis.. Endocr Rev.

[PDF_01934] Baxter J. D. (1976). Glucocorticoid hormone action.. Pharmacol Ther B.

[PDF_01869] Baxter J. D. (1990). Minimizing the side effects of glucocorticoid therapy.. Adv Intern Med.

[PDF_02065] Beisel W. R. (1975). Metabolic response to infection.. Annu Rev Med.

[PDF_02013] Bertagna X., Bertagna C., Luton J. P., Husson J. M., Girard F. (1984). The new steroid analog RU 486 inhibits glucocorticoid action in man.. J Clin Endocrinol Metab.

[PDF_01976] Besedovsky H., del Rey A., Sorkin E., Dinarello C. A. (1986). Immunoregulatory feedback between interleukin-1 and glucocorticoid hormones.. Science.

[PDF_01913] Beutler B., Cerami A. (1987). Cachectin: more than a tumor necrosis factor.. N Engl J Med.

[PDF_01908] Bevilacqua M. P., Pober J. S., Wheeler M. E., Cotran R. S., Gimbrone M. A. (1985). Interleukin 1 acts on cultured human vascular endothelium to increase the adhesion of polymorphonuclear leukocytes, monocytes, and related leukocyte cell lines.. J Clin Invest.

[PDF_01877] Björk J., Goldschmidt T., Smedegård G., Arfors K. E. (1985). Methylprednisolone acts at the endothelial cell level reducing inflammatory responses.. Acta Physiol Scand.

[PDF_02068] Blecher M., Goldstein S. (1977). Hormone receptors: VI. On the nature of the binding of glucagon and insulin to human circulating mononuclear leukocytes.. Mol Cell Endocrinol.

[PDF_01919] Bochner B. S., Rutledge B. K., Schleimer R. P. (1987). Interleukin 1 production by human lung tissue. II. Inhibition by anti-inflammatory steroids.. J Immunol.

[PDF_02050] Bodel P., Dillard G. M., Kaplan S. S., Malawista S. E. (1972). Anti-inflammatory effects of estradiol on human blood leukocytes.. J Lab Clin Med.

[PDF_02042] Bonta I. L., Vargaftig B. B., de Vos C. J., Grijsen H. (1969). Haemorrhagic mechanisms of some snake venoms in relation to protection by estriol succinate of blood vessel damage.. Life Sci.

[PDF_01755] Bucala R., Cerami A. (1992). Advanced glycosylation: chemistry, biology, and implications for diabetes and aging.. Adv Pharmacol.

[PDF_01831] Buschard K., Madsbad S., Rygaard J. (1980). Depressed suppressor cell activity in patients with newly diagnosed insulin-dependent diabetes mellitus.. Clin Exp Immunol.

[PDF_01942] Butler W. T. (1975). Corticosteroids and immunoglobulin synthesis.. Transplant Proc.

[PDF_01993] Casley-Smith J. R., Window J. (1976). Quantitative morphological correlations of alterations in capillary permeability, following histamine and moderate burning, in the mouse diaphragm; and the effects of benzopyrones.. Microvasc Res.

[PDF_01936] Claman H. N. (1972). Corticosteroids and lymphoid cells.. N Engl J Med.

[PDF_01944] Claman H. N. (1975). How corticosteroids work.. J Allergy Clin Immunol.

[PDF_01693] Corkey R. F., Corkey B. E., Gimbrone M. A. (1981). Hexose transport in normal and SV40-transformed human endothelial cells in culture.. J Cell Physiol.

[PDF_01886] Crutchley D. J., Ryan U. S., Ryan J. W. (1985). Glucocorticoid modulation of prostacyclin production in cultured bovine pulmonary endothelial cells.. J Pharmacol Exp Ther.

[PDF_01666] Cuatrecasas P. (1971). Insulin--receptor interactions in adipose tissue cells: direct measurement and properties.. Proc Natl Acad Sci U S A.

[PDF_02075] Cury Y., Garcia-Leme J. (1984). The inflammatory response of hyperthyroid and hypothyroid rats. Role of adrenocortical steroids.. Agents Actions.

[PDF_02000] Davidson F. F., Dennis E. A. (1989). Biological relevance of lipocortins and related proteins as inhibitors of phospholipase A2.. Biochem Pharmacol.

[PDF_01912] Dinarello C. A., Mier J. W. (1987). Lymphokines.. N Engl J Med.

[PDF_01850] Dolkart R. E., Halpern B., Perlman J. (1971). Comparison of antibody responses in normal and alloxan diabetic mice.. Diabetes.

[PDF_01821] Drachman R. H., Root R. K., Wood W. B. (1966). Studies on the effect of experimental nonketotic diabetes mellitus on antibacterial defense. I. Demonstration of a defect in phagocytosis.. J Exp Med.

[PDF_01723] EDLUND T., LOFGREN B., VALI L. (1952). Toxicity of dextran in rats.. Nature.

[PDF_01883] Erlansson M., Svensjö E., Bergqvist D. (1989). Leukotriene B4-induced permeability increase in postcapillary venules and its inhibition by three different antiinflammatory drugs.. Inflammation.

[PDF_01782] Esmann V. (1972). The diabetic leukocyte.. Enzyme.

[PDF_01834] Fairchild R. S., Kyner J. L., Abdou N. I. (1982). Specific immunoregulation abnormality in insulin-dependent diabetes mellitus.. J Lab Clin Med.

[PDF_01938] Fauci A. S., Dale D. C. (1974). The effect of in vivo hydrocortisone on subpopulations of human lymphocytes.. J Clin Invest.

[PDF_01998] Flower R. J. (1986). Background and discovery of lipocortins.. Agents Actions.

[PDF_01812] Fortes Z. B., Farsky S. P., Oliveira M. A., Garcia-Leme J. (1991). Direct vital microscopic study of defective leukocyte-endothelial interaction in diabetes mellitus.. Diabetes.

[PDF_01712] Fortes Z. B., Garcia Leme J., Scivoletto R. (1983). Influence of diabetes on the reactivity of mesenteric microvessels to histamine, bradykinin and acetylcholine.. Br J Pharmacol.

[PDF_01720] Fortes Z. B., Garcia Leme J., Scivoletto R. (1984). Vascular reactivity in diabetes mellitus: possible role of insulin on the endothelial cell.. Br J Pharmacol.

[PDF_01715] Fortes Z. B., Garcia Leme J., Scivoletto R. (1983). Vascular reactivity in diabetes mellitus: role of the endothelial cell.. Br J Pharmacol.

[PDF_01706] Fortes Z. B., Scivoletto R., Garcia-Leme J. (1989). Functional changes in the microcirculation of alloxan-induced diabetic rats.. Gen Pharmacol.

[PDF_01663] Freychet P., Roth J., Neville D. M. (1971). Insulin receptors in the liver: specific binding of ( 125 I)insulin to the plasma membrane and its relation to insulin bioactivity.. Proc Natl Acad Sci U S A.

[PDF_01759] Fussganger R. D., Kahn C. R., Roth J., De Meyts P. (1976). Binding and degradation of insulin by human peripheral granulocytes. Demonstration of specific receptors with high affinity.. J Biol Chem.

[PDF_02025] GLENN E. M., MILLER W. L., SCHLAGEL C. A. (1963). METABOLIC EFFECTS OF ADRENOCORTICAL STEROIDS IN VIVO AND IN VITRO: RELATIONSHIP TO ANTI-INFLAMMATORY EFFECTS.. Recent Prog Horm Res.

[PDF_01898] Gamble J. R., Harlan J. M., Klebanoff S. J., Vadas M. A. (1985). Stimulation of the adherence of neutrophils to umbilical vein endothelium by human recombinant tumor necrosis factor.. Proc Natl Acad Sci U S A.

[PDF_02063] Garcia Leme J., Morato M., Souza M. Z. (1975). Anti-inflammatory action of glucagon in rats.. Br J Pharmacol.

[PDF_01746] Garcia-Leme J., Böhm G. M., Migliorini R. H., de Souza M. Z. (1974). Possible participation of insulin in the control of vascular permeability.. Eur J Pharmacol.

[PDF_01805] Garcia-Leme J., Fortes Z. B., Sannomiya P., Farsky S. P. (1992). Insulin, glucocorticoids and the control of inflammatory responses.. Agents Actions Suppl.

[PDF_02088] Gaunt R., Gisoldi E., Smith N., Steinetz B. G., Chart J. J. (1970). Effect of thyroidectomy and aminoglutethimide on adrenal function in rats.. Endocrinology.

[PDF_01767] Gavin J. R., Buell D. N., Roth J. (1972). Water-soluble insulin receptors from human lymphocytes.. Science.

[PDF_01757] Gavin J. R., Roth J., Jen P., Freychet P. (1972). Insulin receptors in human circulating cells and fibroblasts.. Proc Natl Acad Sci U S A.

[PDF_01951] Gillis S., Crabtree G. R., Smith K. A. (1979). Glucocorticoid-induced inhibition of T cell growth factor production. I. The effect on mitogen-induced lymphocyte proliferation.. J Immunol.

[PDF_02005] Goulding N. J., Guyre P. M. (1992). Regulation of inflammation by lipocortin 1.. Immunol Today.

[PDF_01769] Grunberger G., Robert A., Carpentier J. L., Dayer J. M., Roth A., Stevenson H. C., Orci L., Gorden P. (1985). Human circulating monocytes internalize 125I-insulin in a similar fashion to rat hepatocytes: relevance to receptor regulation in target and nontarget tissues.. J Lab Clin Med.

[PDF_01838] Gupta S., Fikrig S. M., Khanna S., Orti E. (1982). Deficiency of suppressor T-cells in insulin-dependent diabetes mellitus: an analysis with monoclonal antibodies.. Immunol Lett.

[PDF_01855] Gustafsson J. A., Carlstedt-Duke J., Poellinger L., Okret S., Wikström A. C., Brönnegård M., Gillner M., Dong Y., Fuxe K., Cintra A. (1987). Biochemistry, molecular biology, and physiology of the glucocorticoid receptor.. Endocr Rev.

[PDF_01779] Hajek A. S., Joist J. H., Baker R. K., Jarett L., Daughaday W. H. (1979). Demonstration and partial characterization of insulin receptors in human platelets.. J Clin Invest.

[PDF_01691] Haskell J. F., Meezan E., Pillion D. J. (1985). Identification of the insulin receptor of cerebral microvessels.. Am J Physiol.

[PDF_02048] Hempel K. H., Fernandez L. A., Persellin R. H. (1970). Effect of pregnancy sera on isolated lysosomes.. Nature.

[PDF_01671] Häring H. U. (1991). The insulin receptor: signalling mechanism and contribution to the pathogenesis of insulin resistance.. Diabetologia.

[PDF_01871] Inagaki N., Miura T., Nagai H., Ono Y., Koda A. (1988). Inhibition of vascular permeability increase in mice. An additional anti-allergic mechanism of glucocorticoids.. Int Arch Allergy Appl Immunol.

[PDF_01926] Ishikawa H., Mori Y., Tsurufuji S. (1969). The characteristic feature of glucocorticoids after local application with reference to leucocyte migration and protein exudation.. Eur J Pharmacol.

[PDF_02031] Ishioka T., Honda Y., Sagara A., Shimamoto T. (1969). The effects of oestrogens on bluing lesions by bradykinin and histamine.. Acta Endocrinol (Copenh).

[PDF_01740] Joris I., Majno G., Ryan G. B. (1972). Endothelial contraction in vivo: a study of the rat mesentery.. Virchows Arch B Cell Pathol.

[PDF_02054] Klebanoff S. J. (1979). Effect of estrogens on the myeloperoxidase-mediated antimicrobial system.. Infect Immun.

[PDF_01668] Kono T., Barham F. W. (1971). The relationship between the insulin-binding capacity of fat cells and the cellular response to insulin. Studies with intact and trypsin-treated fat cells.. J Biol Chem.

[PDF_01787] Kontras S. B., Bodenbender J. G. (1968). Studies of the inflammatory cycle in juvenile diabetes.. Am J Dis Child.

[PDF_01762] Krug U., Krug F., Cuatrecasas P. (1972). Emergence of insulin receptors on human lymphocytes during in vitro transformation.. Proc Natl Acad Sci U S A.

[PDF_01785] Larkin J. G., Frier B. M., Ireland J. T. (1985). Diabetes mellitus and infection.. Postgrad Med J.

[PDF_02016] Laue L., Kawai S., Brandon D. D., Brightwell D., Barnes K., Knazek R. A., Loriaux D. L., Chrousos G. P. (1988). Receptor-mediated effects of glucocorticoids on inflammation: enhancement of the inflammatory response with a glucocorticoid antagonist.. J Steroid Biochem.

[PDF_01656] Leme J. G. (1981). Regulatory mechanisms in inflammation: new aspects of autopharmacology.. Gen Pharmacol.

[PDF_01962] Leme J. G., Schapoval E. E. (1975). Stimulation of the hypothalamo-pituitary-adrenal axis by compounds formed in inflamed tissue.. Br J Pharmacol.

[PDF_01948] Lew W., Oppenheim J. J., Matsushima K. (1988). Analysis of the suppression of IL-1 alpha and IL-1 beta production in human peripheral blood mononuclear adherent cells by a glucocorticoid hormone.. J Immunol.

[PDF_01867] Litwack G. (1988). The glucocorticoid receptor at the protein level.. Cancer Res.

[PDF_01773] Livingston J. N., Saran B. R., Rose C. D., Anderson C. L. (1985). Rapid effects of insulin on the cycling of the insulin receptor in a human monocyte cell line (U-937).. Diabetes.

[PDF_01727] Llorach M. A., Böhm G. M., Leme J. G. (1976). Decreased vascular reactions to permeability factors in experimental diabetes.. Br J Exp Pathol.

[PDF_01902] Lo S. K., Detmers P. A., Levin S. M., Wright S. D. (1989). Transient adhesion of neutrophils to endothelium.. J Exp Med.

[PDF_01709] Longhurst P. A., Head R. J. (1985). Responses of the isolated perfused mesenteric vasculature from diabetic rats: the significance of appropriate control tissues.. J Pharmacol Exp Ther.

[PDF_01904] Lopez A. F., Williamson D. J., Gamble J. R., Begley C. G., Harlan J. M., Klebanoff S. J., Waltersdorph A., Wong G., Clark S. C., Vadas M. A. (1986). Recombinant human granulocyte-macrophage colony-stimulating factor stimulates in vitro mature human neutrophil and eosinophil function, surface receptor expression, and survival.. J Clin Invest.

[PDF_01732] MAJNO G., PALADE G. E. (1961). Studies on inflammation. 1. The effect of histamine and serotonin on vascular permeability: an electron microscopic study.. J Biophys Biochem Cytol.

[PDF_01639] MILES A. A., MILES E. M., BURKE J. (1957). The value and duration of defence reactions of the skin to the primary lodgement of bacteria.. Br J Exp Pathol.

[PDF_01634] MILES A. A., NIVEN J. S. F. (1950). The enhancement of infection during shock produced by bacterial toxins and other agents.. Br J Exp Pathol.

[PDF_02034] MUELLER M. N., KAPPAS A. (1964). ESTROGEN PHARMACOLOGY. II. SUPPRESSION OF EXPERIMENTAL IMMUNE POLYARTHRITIS.. Proc Soc Exp Biol Med.

[PDF_01841] MacCuish A. C., Jordan J., Campbell C. J., Duncan L. J., Irvine W. J. (1975). Cell-mediated immunity in diabetes mellitus; lymphocyte transformation by insulin and insulin fragments in insulin-treated and newly-diagnosed diabetes.. Diabetes.

[PDF_01704] MacLeod K. M. (1985). The effect of insulin treatment on changes in vascular reactivity in chronic, experimental diabetes.. Diabetes.

[PDF_01889] Maca R. D., Fry G. L., Hoak J. C. (1978). The effects of glucocorticoids on cultured human endothelial cells.. Br J Haematol.

[PDF_01735] Majno G., Gilmore V., Leventhal M. (1967). On the mechanism of vascular leakage caused by histaminetype mediators. A microscopic study in vivo.. Circ Res.

[PDF_01737] Majno G., Shea S. M., Leventhal M. (1969). Endothelial contraction induced by histamine-type mediators: an electron microscopic study.. J Cell Biol.

[PDF_02099] Malaisse W. J., Malaisse-Lagae F., McCraw E. F. (1967). Effects of thyroid function upon insulin secretion.. Diabetes.

[PDF_02096] Mengoli C., Montesi G., Lechi A., Ros A., Bellotti G., Scuro L. A. (1980). Influence of thyroid hormone on cortisol biosynthesis. A gaschromatographic analysis.. J Steroid Biochem.

[PDF_01794] Miller M. E., Baker L. (1972). Leukocyte functions in juvenile diabetes mellitus: humoral and cellular aspects.. J Pediatr.

[PDF_02093] Moore N. A., Boler R. K. (1976). A comparative morphometric analysis of the effects of thyroxine and ACTH on the zona fasciculata of rat adrenal cortex.. Am J Anat.

[PDF_01967] Moraes F. R., Garcia Leme J. (1982). Endogenous corticosteroids and insulin in acute inflammation.. Microvasc Res.

[PDF_01742] Morel N. M., Petruzzo P. P., Hechtman H. B., Shepro D. (1990). Inflammatory agonists that increase microvascular permeability in vivo stimulate cultured pulmonary microvessel endothelial cell contraction.. Inflammation.

[PDF_01895] Moser R., Schleiffenbaum B., Groscurth P., Fehr J. (1989). Interleukin 1 and tumor necrosis factor stimulate human vascular endothelial cells to promote transendothelial neutrophil passage.. J Clin Invest.

[PDF_01792] Mowat A., Baum J. (1971). Chemotaxis of polymorphonuclear leukocytes from patients with diabetes mellitus.. N Engl J Med.

[PDF_01678] Olefsky J. M. (1976). Decreased insulin binding to adipocytes and circulating monocytes from obese subjects.. J Clin Invest.

[PDF_01673] Olefsky J. M. (1975). Effect of dexamethasone on insulin binding, glucose transport, and glucose oxidation of isolated rat adipocytes.. J Clin Invest.

[PDF_01676] Olefsky J. M. (1976). The insulin receptor: its role in insulin resistance of obesity and diabetes.. Diabetes.

[PDF_01765] Olefsky J., Reaven G. M. (1974). The human lymphocyte: a model for the study of insulin-receptor interaction.. J Clin Endocrinol Metab.

[PDF_01789] PERILLIE P. E., NOLAN J. P., FINCH S. C. (1962). Studies of the resistance to infection in diabetes mellitus: local exudative cellular response.. J Lab Clin Med.

[PDF_01940] Parrillo J. E., Fauci A. S. (1979). Mechanisms of glucocorticoid action on immune processes.. Annu Rev Pharmacol Toxicol.

[PDF_02019] Peers S. H., Moon D., Flower R. J. (1988). Reversal of the anti-inflammatory effects of dexamethasone by the glucocorticoid antagonist RU 38486.. Biochem Pharmacol.

[PDF_01802] Pereira M. A., Sannomiya P., Leme J. G. (1987). Inhibition of leukocyte chemotaxis by factor in alloxan-induced diabetic rat plasma.. Diabetes.

[PDF_01929] Perper R. J., Sanda M., Chinea G., Oronsky A. L. (1974). Leukocyte chemotaxis in vivo. II. Analysis of the selective inhibition of neutrophil or mononuclear cell accumulation.. J Lab Clin Med.

[PDF_01680] Pessin J. E., Gitomer W., Oka Y., Oppenheimer C. L., Czech M. P. (1983). beta-Adrenergic regulation of insulin and epidermal growth factor receptors in rat adipocytes.. J Biol Chem.

[PDF_01701] Pfaffman M. A., Ball C. R., Darby A., Hilman R. (1982). Insulin reversal of diabetes-induced inhibition of vascular contractility in the rat.. Am J Physiol.

[PDF_01699] Pillion D. J., Haskell J. F., Meezan E. (1982). Cerebral cortical microvessels: an insulin-sensitive tissue.. Biochem Biophys Res Commun.

[PDF_01828] Pozzilli P., Zuccarini O., Iavicoli M., Andreani D., Sensi M., Spencer K. M., Bottazzo G. F., Beverley P. C., Kyner J. L., Cudworth A. G. (1983). Monoclonal antibodies defined abnormalities of T-lymphocytes in type I (insulin-dependent) diabetes.. Diabetes.

[PDF_02066] Rayfield E. J., Curnow R. T., George D. T., Beisel W. R. (1973). Impaired carbohydrate metabolism during a mild viral illness.. N Engl J Med.

[PDF_02084] STEINETZ B. G., BEACH V. L. (1963). Some influences of thyroid on the pituitary-adrenal axis.. Endocrinology.

[PDF_01808] Sannomiya P., Pereira M. A., Garcia-Leme J. (1990). Inhibition of leukocyte chemotaxis by serum factor in diabetes mellitus: selective depression of cell responses mediated by complement-derived chemoattractants.. Agents Actions.

[PDF_01979] Sapolsky R., Rivier C., Yamamoto G., Plotsky P., Vale W. (1987). Interleukin-1 stimulates the secretion of hypothalamic corticotropin-releasing factor.. Science.

[PDF_01783] Savin J. A. (1974). Bacterial infections in diabetes mellitus.. Br J Dermatol.

[PDF_01922] Schleimer R. P., Freeland H. S., Peters S. P., Brown K. E., Derse C. P. (1989). An assessment of the effects of glucocorticoids on degranulation, chemotaxis, binding to vascular endothelium and formation of leukotriene B4 by purified human neutrophils.. J Pharmacol Exp Ther.

[PDF_01858] Sherman M. R., Stevens J. (1984). Structure of mammalian steroid receptors: evolving concepts and methodological developments.. Annu Rev Physiol.

[PDF_01917] Snyder D. S., Unanue E. R. (1982). Corticosteroids inhibit murine macrophage Ia expression and interleukin 1 production.. J Immunol.

[PDF_02022] Spangler A. S., Antoniades H. N., Sotman S. L., Inderbitizin T. M. (1969). Enhancement of the anti-inflammatory action of hydrocortisone by estrogen.. J Clin Endocrinol Metab.

[PDF_01915] Staruch M. J., Wood D. D. (1985). Reduction of serum Interleukin-1-like activity after treatment with dexamethasone.. J Leukoc Biol.

[PDF_01969] Stenberg V. I., Bouley M. G., Katz B. M., Lee K. J., Parmar S. S. (1990). Negative endocrine control system for inflammation in rats.. Agents Actions.

[PDF_01972] Sternberg E. M., Hill J. M., Chrousos G. P., Kamilaris T., Listwak S. J., Gold P. W., Wilder R. L. (1989). Inflammatory mediator-induced hypothalamic-pituitary-adrenal axis activation is defective in streptococcal cell wall arthritis-susceptible Lewis rats.. Proc Natl Acad Sci U S A.

[PDF_02081] TIMIRAS P. S., WOODBURY D. M. (1955). Adrenocortical function after administration of thyroxine and triiodothyronine in rats.. J Pharmacol Exp Ther.

[PDF_01824] Tan J. S., Anderson J. L., Watanakunakorn C., Phair J. P. (1975). Neutrophil dysfunction in diabetes mellitus.. J Lab Clin Med.

[PDF_01891] Tonnesen M. G., Smedly L. A., Henson P. M. (1984). Neutrophil-endothelial cell interactions. Modulation of neutrophil adhesiveness induced by complement fragments C5a and C5a des arg and formyl-methionyl-leucyl-phenylalanine in vitro.. J Clin Invest.

[PDF_02010] Tsurufuji S., Sugio K., Takemasa F. (1979). The role of glucocorticoid receptor and gene expression in the anti-inflammatory action of dexamethasone.. Nature.

[PDF_02045] Vincent J. E., Bonta I. L., de Vries-Kragt K., Bhargava N. (1971). L'influence des oestrogènes sur la perméabilité de la paroi vasculaire.. Steroidologia.

[PDF_02007] Vishwanath B. S., Frey F. J., Bradbury M., Dallman M. F., Frey B. M. (1992). Adrenalectomy decreases lipocortin-I messenger ribonucleic acid and tissue protein content in rats.. Endocrinology.

[PDF_02078] WALLACH D. P., REINEKE E. P. (1949). The effect of varying levels of thyroidal stimulation on the ascorbic acid content of the adrenal cortex.. Endocrinology.

[PDF_01819] WERTMAN K. F., HENNEY M. R. (1962). The effects of alloxan diabetes on phagocytosis and susceptibility to infection.. J Immunol.

[PDF_02003] Whitehouse B. J. (1989). Lipocortins, mediators of the anti-inflammatory actions of corticosteroids?. J Endocrinol.

[PDF_01865] Yamamoto K. R. (1985). Steroid receptor regulated transcription of specific genes and gene networks.. Annu Rev Genet.

[PDF_02086] ZARROW M. X., HORGER L. M., MCCARTHY J. L. (1957). Atrophy of adrenal gland following thiouracil and vitamin B12.. Proc Soc Exp Biol Med.

[PDF_01845] Zier K. S., Leo M. M., Spielman R. S., Baker L. (1984). Decreased synthesis of interleukin-2 (IL-2) in insulin-dependent diabetes mellitus.. Diabetes.

